# Computational Fluid–Structure Interaction in Microfluidics

**DOI:** 10.3390/mi15070897

**Published:** 2024-07-09

**Authors:** Hafiz Muhammad Musharaf, Uditha Roshan, Amith Mudugamuwa, Quang Thang Trinh, Jun Zhang, Nam-Trung Nguyen

**Affiliations:** 1 Queensland Micro and Nanotechnology Centre, Griffith University, Brisbane, QLD 4111, Australia; hafizmuhammad.musharaf@griffithuni.edu.au (H.M.M.); uditha.thalangamaarachchige@griffithuni.edu.au (U.R.); amith.mudugamuwa@griffithuni.edu.au (A.M.); q.trinh@griffith.edu.au (Q.T.T.); 2 School of Engineering and Built Environment, Griffith University, Brisbane, QLD 4111, Australia

**Keywords:** micro elastofluidics, fluid–structure interaction, computational methods, microdevices, cardiovascular modelling

## Abstract

Micro elastofluidics is a transformative branch of microfluidics, leveraging the fluid–structure interaction (FSI) at the microscale to enhance the functionality and efficiency of various microdevices. This review paper elucidates the critical role of advanced computational FSI methods in the field of micro elastofluidics. By focusing on the interplay between fluid mechanics and structural responses, these computational methods facilitate the intricate design and optimisation of microdevices such as microvalves, micropumps, and micromixers, which rely on the precise control of fluidic and structural dynamics. In addition, these computational tools extend to the development of biomedical devices, enabling precise particle manipulation and enhancing therapeutic outcomes in cardiovascular applications. Furthermore, this paper addresses the current challenges in computational FSI and highlights the necessity for further development of tools to tackle complex, time-dependent models under microfluidic environments and varying conditions. Our review highlights the expanding potential of FSI in micro elastofluidics, offering a roadmap for future research and development in this promising area.

## 1. Introduction

Microfluidics, encompassing the manipulation and control of fluids within networks of channels with typical dimensions ranging from 0.1 µm to 100 µm, offers distinct advantages compared to traditional laboratory-scale techniques. The square-cube law implies that as device dimensions decrease, heat and mass transfer in a microfluidic device can be significantly improved. Microfluidic devices also facilitate faster and more efficient separation processes. Their high surface-to-volume ratio facilitates rapid modifications in fluid dynamics, which is essential for effective separation. Additionally, precise fluid control and integrated functionalities within these devices allow for enhanced reaction kinetics and reduced process steps, making them ideal for applications in biochemical assays, environmental monitoring, and medical diagnostics [1,2,3,4].

Micro elastofluidics is an emerging and promising research field of microfluidics. Micro elastofluidics was first introduced by Nguyen [5] and holds considerable potential for a variety of applications, including particle/cell separation [6,7,8], controlled drug release [9], tuneable optofluidic devices [10], tuneable droplet-size generation [11], capillary flow enhancement [12], mixing [13,14], and the development of fluidic circuits [15,16].

Despite recent advancements in flexible microfluidic devices, such as those designed for wearable applications, phenomena arising from fluid–structure interaction at both molecular and device scales have yet to be fully explored. The field of micro elastofluidics will benefit from substantial advancements in computational fluid–structure interaction (FSI) methods [17,18,19]. These computational techniques are crucial for designing, optimising, and understanding mechanisms that rely on the intricate interactions between fluidic and structural dynamics at the microscale. In micro elastofluidics, these models could also predict the deformation of microstructures and microchannels due to fluid flow and vice versa. In this context, the incorporation of reliable FSI models is crucial for accurately forecasting interconnected physical phenomena. Utilising high-fidelity simulations enables a thorough examination of the specific application, taking into account all relevant scales involved.

**Figure 1:**
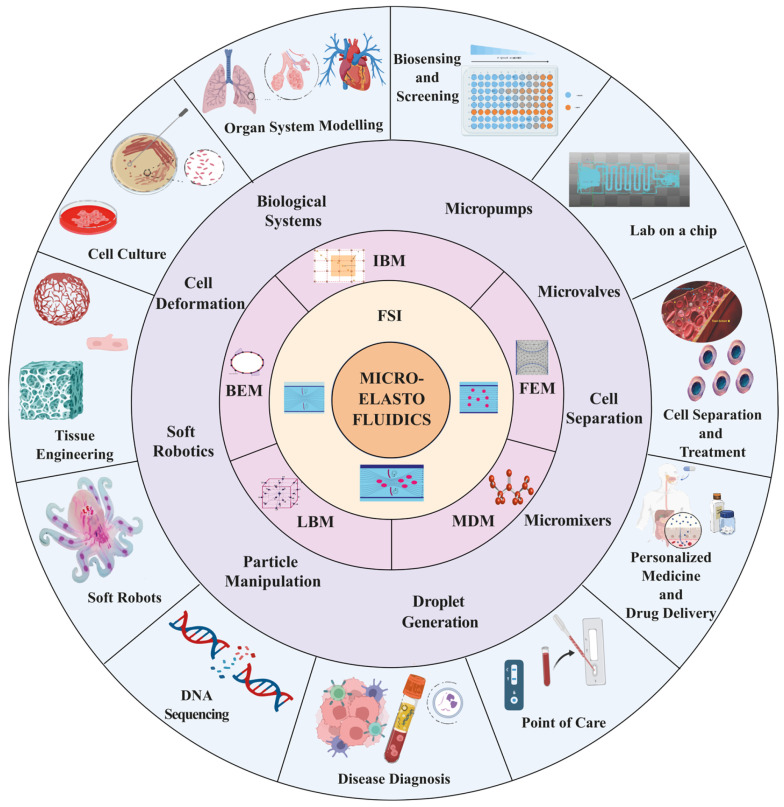
A schematic overview of the computational methods: Finite element method (FEM), boundary element method (BEM), molecular dynamics (MD), lattice Boltzmann method (LBM), and immersed boundary method (IBM) and application domains facilitated by these fluid–structure interaction (FSI) methods.

This review paper highlights the instrumental role of computational methods such as finite element method (FEM), boundary element method (BEM), molecular dynamics (MD), lattice Boltzmann method (LBM), and immersed boundary method (IBM) in advancing FSI for micro elastofluidics. Each of these methods offers distinct advantages and faces specific challenges, making their study and application crucial for the development of microfluidic devices. We also explore the application of computational FSI methods, highlighting their impact on the development of microdevices such as microvalves [20,21], micropumps [22,23,24,25,26], and micromixers [27,28,29,30,31,32,33] (Figure 1). These devices utilise the principles of FSI to enhance their performance and functionality, adapting to the dynamic nature of interactions between fluid flow and elastic structures. Microvalves, for example, benefit from FSI models for optimised flow control, ensuring that they can operate under varying pressures and fluid properties. Micropumps are devices where FSI is indispensable for optimising pump efficiency and reliability under diverse operation conditions.

Moreover, designing micromixers utilises FSI to refine the geometries and mixing mechanisms, enabling effective mixing of fluids at low Reynolds numbers, which are typical in microfluidics. The precise control and enhanced mixing derived from FSI models are vital for applications ranging from chemical synthesis to biological assays.

Beyond device-specific applications, computational FSI methods play a transformative role in biomedical applications such as cell separation [34,35,36,37,38,39] and particle manipulation [40,41,42,43,44,45]. These processes require not only precision but also gentle handling of delicate biological specimens, which can be achieved through well-controlled microenvironment, as facilitated by FSI modelling. In addition, cardiovascular applications of FSI in micro elastofluidics have gained recent attention [46,47,48,49,50]. This research is vital for creating patient-specific cardiovascular applications such as artificial heart valves and on-chip blood analysis systems, which require exact simulations to improve treatment efficacy and patient outcomes [51,52,53,54,55,56]. Moreover, FSI models are essential for designing cardiovascular devices that mimic or interact with the biomechanical properties of tissues, improving therapeutic outcomes and patient-specific treatments. This review also discusses the challenges and future directions in the computational aspect of FSI in micro elastofluidics, including the need for more sophisticated models that can accurately predict complex interactions in real–time and under variable operation conditions. The continued evolution of computational tools and techniques is likely to further enhance the capabilities and applications of FSI in micro elastofluidics, marking an exciting frontier for both fundamental research and practical applications.

Overall, the integration of computational FSI methods into micro elastofluidics represents a significant stride towards more advanced, efficient, and versatile microfluidic systems. Through detailed case studies and theoretical analyses, this review aims to provide a comprehensive overview of the current state and promising future of FSI in this innovative field, encouraging further research and development.

## 2. Fundamentals of Fluid–Structure Interaction in Micro Elastofluidics

In microfluidics, channels and structures with dimensions on the order of micrometres are commonly used to manipulate and control fluid behaviour [57]. Microchannels have been utilised to solve small-scale flow and fluid manipulation tasks within microfluidic devices. Polydimethylsiloxane (PDMS) is a popular material for making microchannels owing to its cost-effectiveness, ease of use, transparency, biocompatibility, and elasticity [4,58,59,60,61]. The low Young’s modulus of PDMS allows for the emulation of blood vessels and soft organs in biomedical studies [62]. Nevertheless, its elasticity poses challenges in both experimental and theoretical modelling due to its susceptibility to a change in channel geometry induced by the flow, and in turn, affecting the overall hydrodynamic behaviour of the device.

In many microfluidic applications, the wall of microchannels is flexible or stretchable to adapt to biological systems, leading to the introduction of the new subfield of micro elastofluidics [5]. Micro elastofluidics is further categorised as digital and continuous-flow micro elastofluidics. Digital micro elastofluidics is based on elastic capsules or deformable beads flowing in the fluid. Continuous micro elastofluidics is based on deformable structures and their interactions with the fluid. Both branches have their unique features and applications. For example, elastic capsules [63] can be used for drug delivery and liquid storage, while stretchable pump [64] is the perfect example of continuous-flow micro elastofluidics.

In micro elastofluidics, unique features of microfluidics such as flow regime, surface tension, diffusion, fluidic resistance, and inertial and shear forces play the same roles in the flow behaviour. Fluid flow is usually laminar in micro elastofluidics since the Reynolds number ($R_{e}=\frac{\rho \upsilon D_{h}}{\mu }$) in most applications is less than one, although high-flow regimes may have *Re* in the range of 10–100. Surface tension, arising from cohesive forces between liquid molecules, is relatively strong within a microfluidic system and can be determined through the Young–Laplace equation ($∆P=\gamma (\frac{1}{R_{1}}+\frac{1}{R_{2}})$) [65,66], where ∆P represents the pressure difference, γ is the surface free energy of the liquid, and $R_{1}$ and $R_{2}$ denote the radii of curvature, perpendicular and parallel to the liquid flow, respectively. Diffusion, the spreading of particles due to random Brownian motion, is determined by the Einstein–Smoluchowski theory ($d^{2}=6Dt$), where d is the distance travelled by the particle, t is time, and D is the coefficient of diffusion. Fluidic resistance (*R*), analogous to electrical resistance, dictates how easily fluids move through microchannels ($Q=\frac{∆P}{R}$), where *Q* is the flow rate and ∆P is the pressure drop across the channel, The fluidic resistance can be calculated using formulas specific to the channel shape [67,68]. However, at small dimensions, such as those encountered in micro/nanochannels, the friction factor undergoes significant changes. While the structural form of the conventional friction factor law is retained, the value of the Poiseuille number increases with the hydrophilicity of the channel walls. This shift highlights the limitations of traditional continuum theories, which fail to account for nanoscale phenomena such as wall slip and spatial variations in transport properties [69]. Due to the flexible and stretchable nature of the channels or particles, an added physics of fluid–structure interaction is necessary to comprehend the fluidic phenomena in the device. In the next section, we discuss how structural deformability interacts with the flow of fluid and vice versa.

### 2.1. Fundamentals of Fluid–Structure Interaction

In micro elastofluidics, the study of FSIs is crucial for understanding the behaviour of fluids confined in flexible microchannels and the impact of this behaviour on the surrounding structures. As described by Duprat and Stone [70], FSI denotes mechanical problems where the flow field affects the orientation, shape, and location of an interacting object, leading to reciprocal modifications in the flow pattern. The flexible and elastic nature of the microchannel does not allow the use of already established rules of rigid microfluidics and necessitates the incorporation of the fluid–structure interaction to obtain suitable engineering solutions.

**Figure 2:**
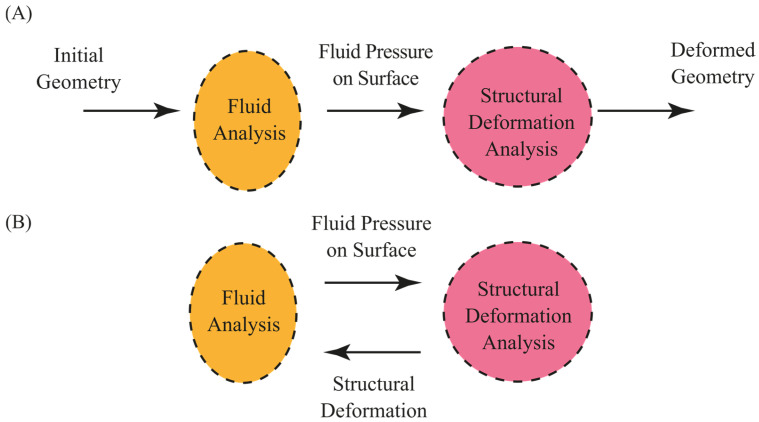
Fluid–structure interaction: (**A**) one-way FSI, (**B**) two-way FSI.

The focus of FSI is on the coupling between fluid dynamics and structural mechanics. Fluid flow exerts forces on a structure, potentially causing it to deform. The magnitude of these deformations depends on fluid pressure, velocity, and material properties of the structure. Minor deformations may not significantly affect the fluid flow. However, larger deformations create a feedback loop where the altered structure modifies the behaviour of the fluid. An FSI with negligible influence of the deformation on fluid flow is considered as a one-way fluid–structure interaction. Problems with one-way FSIs are relatively easy to manage and comprehend. A two-way or fully coupled FSI induces a deformation large enough to affect the fluid flow, which in turn significantly changes the flow-induced deformation of the structure (Figure 2). These complex interactions are important for biomedical problems such as blood flow in flexible vessels [71] and designing medical devices such as micropumps [64,72].

**Figure 3:**
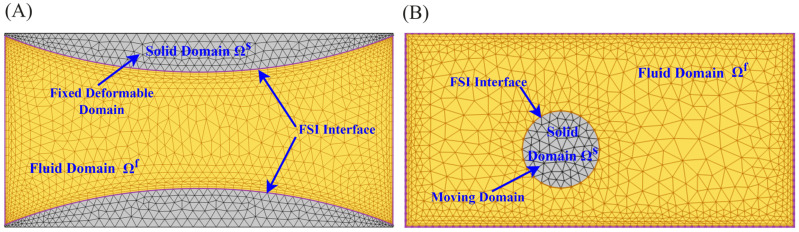
(**A**) Fluid–wall FSI, (**B**) Fluid–particle FSI. Yellow colour shows the fluid domain and grey colour shows the solid domain.

Considering the interface between fluid and a deformable structure, the FSI can also be categorised further into two types: (i) fluid–wall interface and (ii) fluid–particle interface (Figure 3). In FSI with the fluid–wall interface, the deformable structures are the walls of the microchannels (Figure 3A). These walls are elastic, flexible, and fixed. For example, in continuous micro elastofluidics, the walls of the microchannel can be bent and stretched but cannot move. Hence, analysing continuous micro-elastofluidic problems should consider the FSI at the fluid–wall interface. In the FSI at the fluid–particle interface, deformable structures are moving particles (Figure 3B). These particles are elastic and deformable but are not fixed at a point. Fluid forces cause the cell to deform as well as change its position, and these changes in shape and position alter the pattern of fluid flow. For instance, problems related to digital micro elastofluidics are analysed through the FSI at the fluid–particle interface. Methods have been developed to study this FSI at the particle boundary, with applications in cell sorting and biomechanics of cells.

Understanding the relationship between volumetric flow rate Q and pressure drop ∆P is crucial for the design and operation of fluidic systems. In deformable microchannels, this ∆P−Q relationship is nonlinear, which is a major difference from the flow in rigid microchannels [73]. The deformation of channel walls reduces flow resistance, causing a lower pressure drop [19,74]. To elucidate this relationship, numerous experimental works have been conducted, and researchers have developed empirical models for pressure-flow characteristics. For instance, Gervais et al. [73] investigated the behaviour of a fluid flow in stretchable microchannels using confocal microscopy. A model was created to describe the observed pressure-flow characteristics. The results indicated that flexible microchannels could enable higher flow rates compared to rigid microchannels of identical size driven by the same pressure difference. Subsequently, Hardy et al. [75] investigated the behaviour of PDMS microchannels with flexible walls and reported significant differences in pressure drop compared to rigid channels. The team demonstrated that the pressure drop in flexible microchannels decreased to 35% of that in identical channels with rigid walls.

Another FSI problem is compressible flow in deformable channels. The mass flow rate is a function of undeformed microchannel dimensions, the differential pressure across a microchannel, and the characteristics of the channel’s surface such as elastic modulus, thickness, and Poisson’s ratio [19,74,76,77,78,79,80,81,82]. Although efforts have been made toward the development of empirical models for the ∆P−Q relationship, these models only apply to specific conditions with small domain characteristics. To comprehend the generalised and complete FSI phenomena, a numerical analysis is necessary. The next section discusses the fluid dynamics and solid mechanics of FSI and its governing equations.

### 2.2. Fluid Dynamics and Solid Mechanics of FSI

Understanding the fluid dynamics of FSI poses a multifaceted challenge for the design and applications of micro-elastofluidic devices. Key considerations include the dominant role of surface tension, potential hyperelastic responses of the elastomeric materials, and the two-way dynamic coupling between fluid stresses and structural deformations. Analytical solutions are rarely feasible for FSI problems, and lab experiments cannot capture the full range of behaviours. These bottlenecks make numerical simulations crucial for understanding the complex physics of how fluids and solids interact. Understanding the FSI in microfluidics has been advanced through the development of dimensionless continuum finite approaches (FEM, BEM) and digital finite approaches (MDM and LBM) [83]. These methods are discussed thoroughly in the next section.

FSI involves the fluid dynamics as well as solid mechanics of stretchable solids containing the fluid or surrounded by the fluid. Without the effect of the flexible structure on the flowing fluid, the behaviour of the fluid is governed by the equation of continuity:

(1) ∇·u=0

and Navier–Stokes equation:

(2) $$\rho \left(\frac{\partial u}{\partial t}+\left(\nabla .u\right)u\right)=-\nabla p+\mu \nabla^{2}u+F$$

where ρ, u, p, μ are the fluid density, vector velocity, pressure, and dynamic viscosity respectively, t is the time, and F represents the external body force.

The body force F in FSI is introduced by the flexible structure. This force can be applied externally on the deformable structure to simplify the problem in one-way FSIs. Alternatively, this force can be induced in the structure by the flowing fluid in coupled FSIs, hence making the problem more precise at the expense of complexity. This induced force depends on the nature of the material and the behaviour of the flexible structure under stress and strain.

In the solid domain, the equations of motion are typically described by the linear elasticity equations, assuming small deformations. The governing equation is the linearised form of the momentum balance equation [84]:

(3) $$\rho_{s}\frac{\delta^{2}d_{s}}{\delta t^{2}}=\nabla .\sigma$$

where $\rho_{s}$ is the density of the solid, $d_{s}$ represents the displacement of the solid, and σ is the stress tensor.

The constitutive relation between stress and strain (Hooke’s law) is often utilised, linking stress σ to strain ϵ via Lamé’s equation of deformation [85]:

(4) $$\sigma =\lambda t_{r}\left(\epsilon \right)I+2\mu \epsilon$$

where $\lambda =\frac{Eʋ}{\left(1+ʋ)(1-2ʋ\right)}$ is Lamé’s first parameter, representing the material’s compressibility since ʋ is the Poisson’s ratio of the material, $t_{r}\left(\epsilon \right)$ is the trace of the strain tensor ϵ, which corresponds to the volumetric strain, $\mu =\frac{E}{2(1+ʋ)}$ is Lamé’s second parameter, also known as the shear modulus, characterising the material’s resistance to deformation, and I is the identity tensor. The term $\lambda t_{r}\left(\epsilon \right)I$ accounts for the isotropic (volumetric) deformation. This term is then multiplied by the identity tensor I, indicating that this part contributes to the isotropic stress or hydrostatic pressure component. The term 2μϵ represents the deviatoric deformation, accounting for the deformation that leads to shape changes without altering the volume.

The overall equation combines these two components to describe the complete stress tensor σ in the context of fluid–structure interaction. It reflects the material response to both isotropic and deviatoric deformations induced by external forces or fluid interactions. Modelling FSIs in micro-elastofluidic devices, where fluid flow deforms elastomeric structures, requires specialised approaches. Hyperelastic material models, capable of handling large deformations, are to be considered for accurate results. Many constitutive models have been formulated to characterise the nonlinear mechanical behaviour of hyperelastic materials. Within this theoretical framework, the neo-Hookean [86] and Mooney–Rivlin [86] models stand out as widely adopted approaches for describing large deformations, which are characteristic of elastomers.

### 2.3. Boundary Conditions

**Figure 4:**
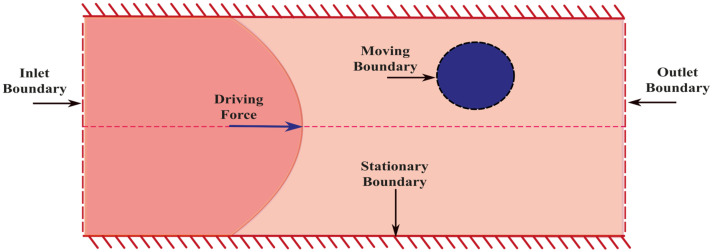
Boundaries in typical FSI problem.

In FSI problems, boundary conditions define the constraints and interactions that shape the behaviour of the system. Three distinct types of boundaries are considered for micro-elastofluidic applications: (i) boundary conditions at the device surface that may be moving in stretchable microfluidics; (ii) boundary conditions related to the surface of particles that also may be in motion and potentially changing shape; (iii) inlet and outlet conditions, due to the open-system nature of microfluidic devices, (Figure 4).

In most cases, device surfaces are considered impermeable and under the no-slip condition. These surfaces are typically regarded as rigid and stationary. Thus, the interaction between the device and the flowing fluid is primarily defined by the static nature of the channel wall and the no-slip condition. However, device walls are deformable in flexible microchannels. Thus, boundary conditions at the device surfaces change with time. We can incorporate these changes in FSI simulations as a moving boundary condition.

The study of elastic particle transport requires the modelling of the FSI at particle boundaries. These interactions encompass both velocity and stress continuity. The movement and alteration of particles impact the flow and are reciprocally influenced by it, thereby establishing an FSI problem. Hydrodynamic forces and the torque on particles determine the translation, rotation, and deformation of the particle surface. These phenomena on particles are studied under Inertial Particle Microfluidics (IPMF). The force and torque acting on particles are calculated as:

(5) F=∮dAσ.n

(6) $$T=\oint dAx\times \left(\sigma .n\right)$$

In these equations, σ, n, and x represent the fluid stress tensor, unit vector normal to the surface, and the spatial position of a point on the surface of the particle, respectively.

Probably the most difficult part of IPMF is numerically solving the FSI problem. Additional forces may become noticeable as particles move close to each other or close to the device’s surface. These forces either have chemical electrostatic interactions, adsorption phenomena, surface chemistry effects, and chemical gradients or physical origins (inertial and viscous drag forces, along with lift and Dean forces). Physical factors such as gravitational and buoyancy forces become insignificant in IPMF because of the high fluid stress and low volume [87]. For considering the elastic behaviour of particles in IPMF, a suitable elastic constitutive model must be selected to describe the particle’s deformation under hydrodynamic stresses.

The inlet and outlet conditions hold significant importance for modelling the fluid–structure interaction in micro elastofluidics. In reality, where only specific parts of a device are pertinent or feasible for simulations, it is crucial to define the flow at the planes of the inlet and outlet of the selected variable. For subsets representing straight channels or unit cells with periodic characteristics such as serpentine channels, periodic boundary conditions usually provide the best and most direct solution. Any particle or liquid leaving one side of the numerical domain enters again into the other side. Essentially, the model is an unlimited collection of unit cells, with the stimulated domain serving as the definition of each unit cell.

On the other hand, periodic boundary conditions are not suitable for subsets with intricate shapes. In such cases, pressure and volume conditions are important at the inlet and outlet. For an unknown flow field at the inlet plane, modellers use a velocity profile to characterise the flow within a specified channel geometry, which presumes no upstream perturbations. For a variety of geometrically simple cross sections, time-independent closed-form solutions of Navier–Stokes equations for duct pipes are available [88], making them an obvious choice for creating a comprehensive velocity profile.

Since flow within the stimulated domains determines the outlet plane flow field, and flow perturbations upstream outside of the domain are minimal, it becomes easier to control the outlet. An alternative outflow condition, often used instead of periodicity, is a zero-gradient condition [89]. Additionally, non-periodic boundary conditions introduce complexities in handling particles entering and exiting the subset.

### 2.4. Coupling Approaches

The selection of a suitable coupling approach is the cornerstone of a successful FSI simulation. This determines the mechanism by which the fluid and structure domains exchange displacement and force information at the interface during iterative solutions or time steps. The selected coupling strategy can significantly impact the numerical stability, convergence rate, and the ability to capture the true physics of the coupled system.

**Figure 5:**
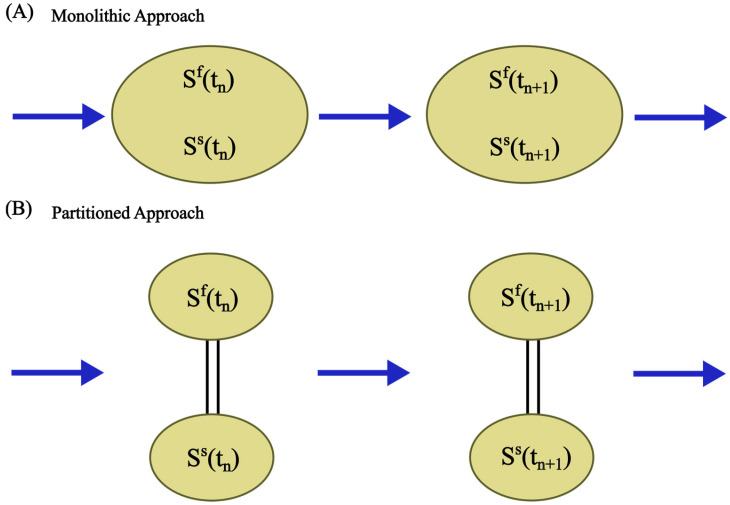
FSI coupling approaches, (**A**) monolithic, (**B**) partitioned.

Two primary approaches exist: monolithic and partitioned (Figure 5). In monolithic approaches, the governing equations for fluid and solid domains are integrated into a single, unified framework and solved simultaneously. Interfacial boundary conditions (e.g., no-slip, stress continuity) are implicitly embedded within this framework. Monolithic schemes offer superior accuracy for tightly coupled FSI problems but often demand the development of highly specialised solvers, potentially increasing implementation complexity and computational expense [90,91,92]. In contrast, the partitioned approach considers the fluid and structure as separate computational domains. This approach enables the independent discretisation of each domain and the application of numerical solution techniques optimised for the governing physics within each domain. The approach allows for the use of specialised, potentially pre-existing “legacy” solvers optimised for each respective physical domain (e.g., fluid vs. structural mechanics). Interfacial data exchange (displacements, tractions) occurs iteratively, potentially simplifying development time and enhancing flexibility. However, careful attention must be paid to interface tracking and the stable, accurate transfer of data across this dynamic interface. Specialised algorithms designed for moving boundary problems are often required to ensure numerical stability and prevent unphysical solution behaviour.

**Figure 6:**
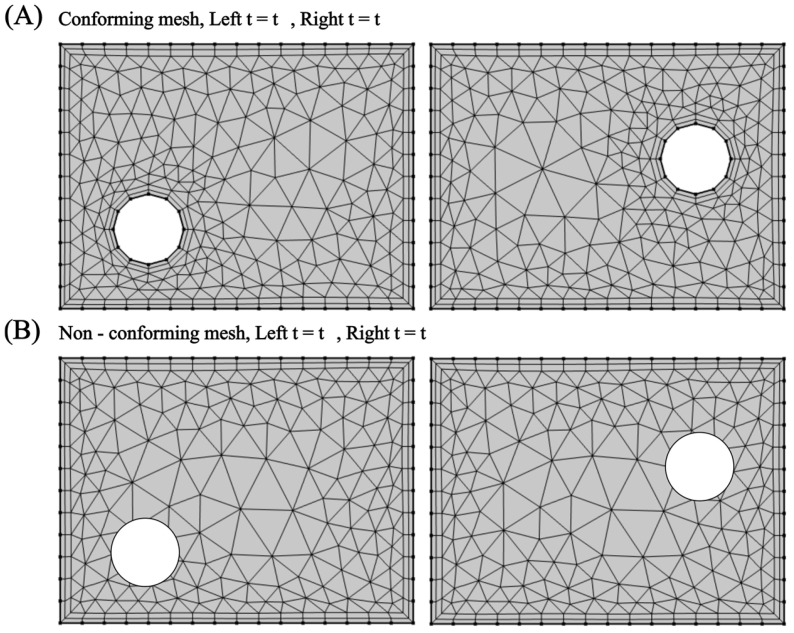
Mesh discretisation approaches, (**A**) conforming meshing, (**B**) non-conforming meshing. White circle and grey area represent the regid body and fluid domain respectively. In conforming meshing scheme, mesh gets updated after every time step while in non-conforming meshing scheme, mesh remains the same.

An additional classification scheme for the FSI solution is based on the discretisation of the mesh, which dictates how interfacial boundary conditions are imposed within the discretised system of equations (Figure 6). One is the conforming meshing method, where the fluid–structure interface is considered as a physical boundary. The fluid and solid domain meshes must perfectly match. This often leads to re-meshing (or mesh-updating) as the structure deforms throughout the simulation, hence adding computational cost. The other is the non-conforming meshing method, where the fluid–structure interface is not treated as a strict alignment boundary. Instead, the interface location and its conditions (e.g., stress continuity) are considered constraints within the governing equations. This approach enables independent meshing for each domain, streamlining the simulation setup and avoiding re-meshing.

## 3. Computational Methods for Studying Fluid–Structure Interactions

**Figure 7:**
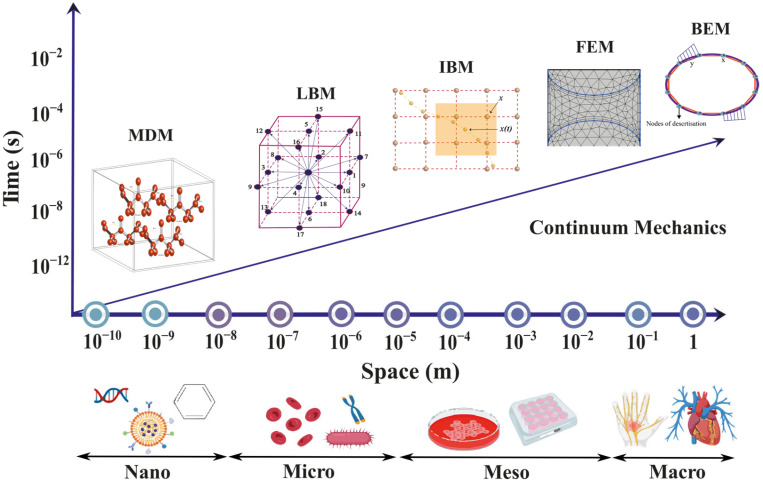
Space and time scale of different computational methods with comparative sizes.

Numerical simulation techniques play a crucial role in science and engineering, enabling researchers to study complex phenomena and make predictions based on mathematical models. In recent years, computational methods have played a significant role in studying FSIs, providing valuable insights into the behaviour of fluids and structures. In the field of micro elastofluidics, numerical methods have been used in the design, analysis, and optimisation of micro-elastofluidic devices. Additionally, numerical approaches such as FEM, BEM, MD, and LBM allow for the discretisation and solving of the governing equations of fluid dynamics and hyperelasticity with their intricate coupling at the fluid–structure interface. These methods are indispensable for predicting flow patterns, understanding how microstructures deform under fluid stresses, and designing novel micro-elastofluidic devices. Notably, these numerical approaches enable the exploration of a wide parameter space far beyond what may be accessible experimentally, aiding the optimisation and development of devices for applications ranging from biomedical diagnostics to soft micro-robotics. Furthermore, the application of these numerical methods extends to various fields, including the development of passive microvalves [93,94], and micromixers [95,96,97], to understand the fluid dynamics and to optimise the performance of these devices. Extensive numerical methods have been developed and refined for microfluidic applications such as biosensors [98,99] and biofluid handling [100] in wearable devices. However, the emerging field of micro elastofluidics warrants further advancements due to the inherent complexities of fluid–structure interactions at the microscale. Therefore, numerical methods are integral to gaining insights into the complex fluid–structure interactions and optimising the performance of micro-elastofluidic systems. This section discusses comprehensively all numerical methods that can be used for FSIs in micro elastofluidics. Figure 7 illustrates the different computational methods in space and time scale.

### 3.1. Finite Element Method

**Figure 8:**
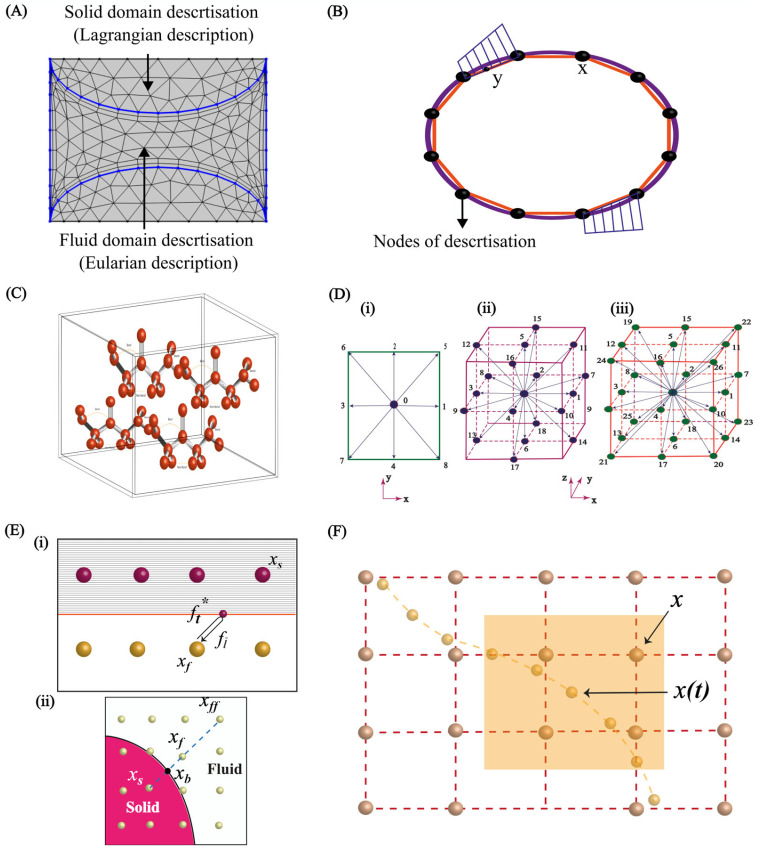
Computational methods for FSIs. (**A**) Discretisation of fluid and solid domain in the FEM; (**B**) discretisation of domain in the BEM; (**C**) molecules’ interaction in the MD method [106]; (**D**) 2D and 3D configurations of the LBM, (**i**) 2D nine velocity configuration, (**ii**) 3D nineteen velocity configuration, (**iii**) 3D twenty seven velocity configuration [107]; (**E**) (**i**) bounce back method [87], (**ii**) extrapolated bounce back method illustration [107]; (**F**) IBM as a Lagrangian and Eulerian description [87].

The finite element method (FEM) is an indispensable computational tool for tackling the complex challenges presented by FSIs in micro elastofluidics. This method divides the domain of interest into smaller elements, allowing for the accurate modelling of complex geometries and boundary conditions (Figure 8A). FEM has been widely applied in various aspects of microfluidics. Hung et al. [101] modelled mass transfer using the FEM in a high-aspect-ratio microfluidic device, which provided a stable and uniform microenvironment for cell growth in a high-throughput mammalian cell culture array. Erickson et al. [102] investigated the role of surface heterogeneity on electrokinetically driven microfluidic with a 3D FEM, aiming to enhance mixing in a T-shaped micromixer. Bianchi et al. [103] implemented FEM models to simulate the electroosmotic-driven flow division at a T-junction.

The FEM has also been applied in various contexts to investigate fluid–structure interactions in microfluidics. For example, Zhang et al. [104] reported a groundbreaking application of the Cell-based Smoothed Finite Element Method (CS-FEM) to computational fluid dynamics (CFD) and FSI simulations. The CS-FEM belongs to the broader family of Smoothed Finite Element Methods (S-FEMs), which aim to enhance accuracy and robustness by applying strain smoothing techniques. Specifically, the CS-FEM simplifies calculations by not requiring explicit shape functions and demonstrates better tolerance to distorted meshes. This study demonstrated that the CS-FEM offered greater accuracy and stability in handling deformable interfaces and flow fields that typify FSI problems, making it well-suited for simulations relevant to micro elastofluidics. Erickson et al. [105] applied the same approach to investigate how smart one-way microvalves behaved when the FSI was taken into account. Hence, the FEM has been proven vital in comprehending FSIs in micro elastofluidics.

A typical FEM approach consists of five steps: (i) Discretisation: the continuum domain is discretised into a finite number of smaller elements that are easier to manage. (ii) Element equation formulation: For each element, the governing physical equations are formulated. These equations typically stem from fundamental conservation laws (such as mass, momentum, and energy conservation) and are expressed in terms of local element variables. (iii) Assembly: The local element equations are assembled into a global system of equations that models the entire problem domain. This step involves integrating the contributions of individual elements to the overall behaviour of the system. (iv) Solution: The global system of equations is solved numerically to find the unknowns, such as displacements and pressures. This solution process may incorporate iterative methods and solvers that handle nonlinearities and complex boundary conditions. (v) Post-processing: The solution obtained is interpreted in terms of physical quantities of interest, such as stress distributions, fluid velocities, and pressures. This helps in evaluating the performance and safety of the micro-elastofluidic devices. The FEM for simulating FSIs in micro elastofluidics consists of modelling the fluid domain, modelling the deformable structure domain, and then the fluid–structure interface coupling. In every phase, specific mesh strategy and physics are used to simulate and then coupled them. Here, we discuss the physics and governing equations of these domains.

#### 3.1.1. Modelling of Fluid Domain

The FEM begins by discretising the fluid domain (e.g., a microfluidic channel) into a mesh of smaller, interconnected elements. These elements are often triangles or quadrilaterals in 2D and tetrahedra or hexahedra in 3D. In this domain, the FEM describes the mesh as an Eulerian space. The behaviour of the fluid within this mesh is governed by the fundamental Navier–Stokes equations. These equations express the conservation of mass, i.e., Equation (1), and momentum, i.e., Equation (2). To solve the Navier–Stokes equations numerically, the FEM employs a technique called the Galerkin method. Essentially, the equations are transformed into their “weak form” and approximated using shape functions. These shape functions describe how the fluid velocity and pressure vary within each element of the mesh. The introduction of shape functions in simulations is defined as:

(7) $$u\left(x\right)=\sum N_{i}(x)u_{i}$$

(8) $$p\left(x\right)=\sum M_{j}p_{j}$$

where $N_{i}(x)$ is the shape function, and $u_{i}$ is the nodal velocity. Similarly, in Equation (8), $M_{j}$ is the shape function, and $p_{j}$ is the nodal pressure.

#### 3.1.2. Modelling of Deformable Structure

The versatility of the FEM extends to handling the solid mechanics of deformable structures interacting with the flow. The FEM divides the structural domain in the same way as the fluid domain, i.e., into small finite elements. However, the structural domain uses a Lagrangian description. The governing equations of solid mechanics, i.e., Equation (9), describe the balance of forces within the structure and the relationship between stress and strain (deformation). The choice of a constitutive model (e.g., linear elastic, hyperelastic, viscoelastic) dictates how the material responds to these stresses. Similar to the fluid domain, FEM discretises deformable structures with elements and uses shape functions to approximate the displacement field.

(9) $$\nabla .\sigma +f_{s}=0$$

(10) $$d\left(x\right)=\sum X_{i}d_{i}$$

where σ is the stress tensor, and $f_{s}$ is body force on the structure. More importantly, this body force comes from the fluid pressure on the structure in FSI problems. In Equation (10), $d\left(x\right)$ is the displacement vector, $X_{i}$ is the shape function, and $d_{i}$ is the nodal displacement vector. For micro elastofluidics, selecting the appropriate shape functions is crucial and directly depends on the material models used to represent the deformable structures. For materials exhibiting linear elastic behaviour, where deformations are small, linear or quadratic shape functions often provide sufficient accuracy. However, microfluidic systems often involve significant deformations, demanding the use of hyperelastic material models. These models describe nonlinear stress–strain relationships, thus necessitating higher-order shape functions within the FEM to accurately represent the complex deformation patterns that can occur.

### 3.2. Boundary Element Method

The boundary element method (BEM) is another numerical technique for solving problems involving FSI in microfluidics. Analysing the behaviour of fluids and structures at the boundaries of microfluidic devices is one of the advantages of the BEM. Unlike the FEM, which divides the entire domain into smaller elements, the BEM is based on discretising the boundary of the domain of interest as shown in Figure 8B.

For FSIs in micro elastofluidics, the BEM converts the governing partial differential equations (PDEs) of fluid dynamics and structural mechanics into boundary integral equations using appropriate Green’s functions [108]. The integral equations for both the fluid and structural domains are discretised using boundary elements. The interactions are numerically integrated across each element. This step involves the computation of governing coefficients, which quantify the effect of one element on another across the fluid–structure interface. The discretised equations form a system of linear algebraic equations, which are solved to ascertain the unknown boundary values, such as fluid pressures and structural displacements. This resolution often employs iterative methods, especially when the interaction between fluid and structure exhibits significant nonlinearity. After computing the boundary values, further calculations are conducted to evaluate other internal fields as required. For micro elastofluidic devices, this may involve determining fluid velocity field or stress distribution within the structural material, vital for the thorough analysis and design of the devices.

The formulation of integral equations in BEM is crucial for solving problems involving FSIs in micro elastofluidics. Integral equations are derived from the domain’s governing differential equations using mathematical techniques involving Green’s functions. Green’s functions are specific solutions to differential equations under impulse (or point source) conditions and are fundamental to problems in physics and engineering. Essentially, these functions describe how effects such as stress, heat, or electromagnetic fields propagate from a source point to an observation point in space.

The BEM uses Green’s functions to convert the local description of the phenomena (differential equations) into a global one (integral equations) over the boundary. This conversion is based on boundary integral theorems such as Green’s theorem in potential theory or Kelvin’s theorem in elasticity [109], which relates the values of a function inside a domain to values on the domain’s boundary. The specific Green’s function depends on the type of differential equation and the nature of the domain (e.g., infinite space, half-space, bounded domain). Once the appropriate Green’s function is selected, the integration over the boundary can be set up. This step is crucial because it reduces the problem from a volumetric one to a surface-based one, significantly simplifying the computational domain and potentially reducing the amount of computational effort required. However, accurately computing Green’s functions and their integrals over complex boundary shapes can be mathematically and numerically challenging, often requiring sophisticated numerical integration techniques and careful handling of singularities.

For fluids, particularly under the assumption of potential flow, which is common in many microfluidic applications due to the low Reynolds number, the fluid behaviour can be described by the Laplace equation for the potential ∅, where $\nabla^{2}\emptyset =0$. The integral equation for the fluid potential, derived from the Laplace equation, is expressed as [108]:

(11) $$\emptyset \left(x\right)=\int_{\Gamma }^{.}\emptyset \left(y\right)\frac{\partial G(x,y)}{\partial n_{y}}dS_{y}-\int_{\Gamma }^{.}G(x,y)\frac{\partial \emptyset \left(y\right)}{\partial n_{y}}dS_{y}$$

where $\;\emptyset \left(x\right)$ is the potential at the point $\left(x\right)$, G(x,y) is Green’s function for the Laplacian, applicable to the boundary conditions and geometry of the fluid domain, Γ denotes the boundary of the domain,$\;\frac{\partial }{\partial \left(n_{y}\right)}$ represents the derivative normal to the boundary at point y, $dS_{y}$ is the differential boundary element at y. This equation effectively transforms the volumetric problem of fluid dynamics into a surface problem, simplifying the computational domain to just the boundaries where fluid and structural interactions occur.

For the structural component, when considering elastic behaviour under small deformations, the displacement field d in the structure can be described using linear elasticity. Assuming isotropic and homogeneous material properties, the corresponding integral equation derived from the Navier–Cauchy equations for elasticity is [108]:

(12) $$d\left(x\right)=\int_{\Gamma }^{.}T\left(y,x\right)d\left(y\right)dS_{y}-\int_{\Gamma }^{.}D\left(y,x\right)t(y)dS_{y}$$

where $d\left(x\right)$ is the displacement at point x,$\;T\left(y,x\right)$ and $D\left(y,x\right)$ are the traction and displacement kernels derived from the fundamental solutions of elasticity, and t(y) represents the traction at point y on the boundary Γ.

Coupling at the fluid–structure interface requires that the fluid forces, e.g., pressure and shear stress derived from the fluid potential ∅ and the structural responses such as displacements and stresses, accurately match at the interface. Kinematic continuity ensures that the displacement of the structure matches the fluid displacement at the boundary. Dynamic continuity ensures that the sum of stress vectors from both fluid and structure at their interface is zero, maintaining the force balance. This is achieved by ensuring the continuity of displacements and tractions across the interface, linking the fluid and structural integral equations. These coupled equations are then solved iteratively or simultaneously to yield the interaction dynamics essential for device functionality. This coupled approach, facilitated by the BEM, allows for a sophisticated analysis of interactions in micro-elastofluidic devices, providing critical insights into device performance and guiding design optimisations. Through precise mathematical formulations and boundary-focused computations, the BEM offers a powerful solution to complex FSI challenges in the field of micro elastofluidics.

The BEM offers several advantages for FSIs in micro elastofluidics. The BEM is particularly advantageous for problems defined on unbounded domains, as it only requires the discretisation of the boundary where the solution is sought. This makes the BEM well-suited for problems where the solution behaviour is primarily on the boundary or exterior of the domain, such as in potential flow problems, acoustics, and some electromagnetic problems. The BEM potentially requires fewer elements and computational resources compared to volumetric discretisation methods, leading to a reduced computational effort. However, the boundary element method also has limitations. The BEM can face challenges in dealing with internal singularities within the domain, requiring additional techniques or modifications to accurately handle such cases. Furthermore, the accuracy of BEM can cause errors in boundary conditions that may significantly affect the solution. Despite the reduced domain discretisation, BEM can become complex and computationally demanding for highly irregular or complex geometries.

In the context of microfluidics, BEM has been utilised to model and analyse fluid–structure interactions. For example, Martinez et al. [110] demonstrated the use of the BEM for prototyping paper-based microfluidic designs. Li et al. [111] also utilised the BEM to create paper-based microfluidic devices by plasma treatment, showcasing the method’s versatility in creating microfluidic devices. Additionally, Everstine et al. [112] developed a coupled finite element/boundary element approach for FSIs, demonstrating the capability of the BEM in addressing complex FSIs.

### 3.3. Molecular Dynamics Method

Molecular dynamics (MD) simulations are a potent computational method for studying the behaviour of microfluidics at the molecular level [113,114,115,116]. In MD simulations, positions and velocities of individual atoms or molecules are tracked over time by the numerical integration of Newton’s equations of motion (Figure 8C). This allows for the investigation of the dynamic behaviour and interactions of the fluid and structure constituents in microfluidic devices and systems.

Theoretically, the MD method includes all reactions and forces happening at the atomistic level and then integrates these effects at the global level and defines the fluid properties and states. For example, for a fluid consisting of N interacting atoms or molecules generally represented as particles, each characterised by positions $x_{i}$ and velocities $v_{i}$, where 1≤i≤N, the Newton equation of motions is as follows [117]:

(13) $$\frac{d}{dt}x_{i}=u_{i}$$

(14) $$\frac{d}{dt}v_{i}=\frac{1}{m_{i}}\nabla_{i}V(x_{1},\ldots ..,x_{N})$$

where 1≤i≤N.

The individual velocities are derived from Hamiltonian dynamics [118]. The Hamiltonian represents the total energy of the system, encapsulating both kinetic and potential energy components. The Hamiltonian (H) of a molecular system in MD simulations is defined as the sum of the kinetic energy (T) of the particles and the potential energy (V) arising from their interactions as [118]:

(15) $$H=T+V=\sum_{i=1}^{N}\frac{\left|p_{i}\right|}{2m_{i}}+V\left({\vec{r}}_{1},{\vec{r}}_{2},\ldots ,{\vec{r}}_{N}\right)$$

where  N is the number of particles, $p_{i}$ is the momentum of the ith particle, $r_{i}$ is its position, and $m_{i}$ is its mass. Further, the momentum $p_{i}$ is related to the velocity $u_{i}$ of the particle by ${p_{i}=m}_{i}u_{i}$. The potential energy V represents all the interactions between particles. These interactions can be intramolecular (bond stretching, angle bending, dihedral angles) and intermolecular (van der Waals forces, electrostatic forces). The specific form of V depends on the model and the nature of the forces considered, often derived from quantum mechanical calculations or empirical data. Hamiltonian dynamics inherently conserves the total energy, as well as other quantities like linear momentum and angular momentum, assuming no external forces acting on the system.

Due to the nature of being a system of ordinary differential equations, the integration process of Equation (14) is numerically relatively simple. The ease of numerical integration is a key factor that contributed to the widespread utility and popularity of MD as a valuable simulation method for various problems. However, in scenarios where a fluid spans macroscopic length scales, computational modelling solely with MD is currently unattainable due to the substantial number of particles present in a macroscopic fluid section. Given the impracticality of MD for such scenarios, it is often more convenient to approximate the behaviour of the fluid as a continuum rather than a collection of individual particles. This continuum description can be derived directly from Equation (14). Considering the probability density function $f\left(\left\{x_{i}\right\},\left\{v_{i}\right\},t\right)$ representing the positions and velocities of the N particles. The continuum mass density and velocity fields of the fluid are then defined, respectively, as [117]:

(16) $$\rho \left(x,t\right)=\sum_{k=1}^{N}m_{k}\int dx_{1},\ldots \ldots ,dx_{N}dv_{1},\ldots \ldots ,dv_{N}\delta \left(x-x_{k}\right)f\left(\left\{x_{i}\right\},\left\{v_{i}\right\},t\right)$$

and

(17) $$u\left(x,t\right)=\frac{1}{\rho \left(x,t\right)}\sum_{k=1}^{N}m_{k}\int dx_{1},\ldots \ldots ,dx_{N}dv_{1},\ldots \ldots ,dv_{N}v_{k}\delta \left(x-x_{k}\right)f\left(\left\{x_{i}\right\},\left\{v_{i}\right\},t\right)$$

It is important to note here these fields such as mass density, velocity, and pressure are connected to statistical average microscopic densities of these quantities, reflecting a statistical average over a large number of molecules. Therefore, the continuum approximation used in fluid dynamics can be seen as a moment or mean-field approximation, where macroscopic properties are derived from the averaged effects of microscopic interactions. This approach bridges the gap between molecular-level behaviours captured by MD simulations and the macroscopic descriptions typical of continuum mechanics [106,119,120]. The accuracy and reliability of MD simulations depend on the choice of force fields and simulation parameters [121,122,123,124,125,126,127]. Force fields describe the interatomic or intermolecular interactions and are critical for obtaining accurate results. These forces involve Lennard-Jones potentials for van der Waals forces, Coulombic potentials for electrostatic forces, and specific bonded interactions (stretching, bending, and torsional forces for polymers) [126,127].

At the microscale, the combined effects of viscosity and elasticity significantly influence fluid behaviour, resulting in distinct properties that are critical for microfluidic applications. Enhanced viscosity at that scale affects the fluidic resistance, while altered elasticity impacts how materials respond to deformation and stress [128]. These characteristics profoundly influence the behaviour of fluids under mechanical stress and deformation, crucial for optimising microfluidic device performance. Several works have been performed to study the elastic and viscous properties of the fluid at the micro- and nanoscale using MD simulations. For instance, Liu et al. [129] conducted MD simulations to investigate the mechanical properties of polymer nanocomposites. The simulation provided insights into the effect of nanoparticle size, concentration, and surface chemistry on the elastic modulus and viscosity of the composite material. Transport properties and fluid flow behaviour in microfluidics have been studied by MD simulations. For instance, Wang et al. [130] used MD simulations to investigate the flow of polymer solutions through microchannels. The authors analysed the effects of polymer concentration, molecular weight, and channel geometry on flow behaviour and observed the formation of elastic instabilities. Modi et al. [106] studied the elastic properties of polypropylene and cellulose nanofibrils bionanocomposite systems using MD simulations and revealed that the amount of cellulose had strong influence on elastic properties of the system. This MD investigation also predicted that the introduction of maleic anhydride as the coupling agent could improve the elastic modulus by changing the hydrophobic–hydrophilic adhesion between the components of the bionanocomposite.

MD simulations also enabled the study of behaviour of biological systems. Zhang et al. [131] performed MD simulations to investigate the mechanical properties of red blood cells (RBCs) in microchannels. The authors studied the deformation and flow behaviour of RBCs under various conditions and observed the formation of cell-free layers and cell aggregation. Nair et al. [132] stated that MD simulations could be used to study the protein structure–function relationships and had great potential application in the field of drug delivery.

MD simulations can provide valuable insights into the elastic and viscous properties of fluids at the molecular level [106,133]. They allow for the study of complex phenomena and provide an in-depth understanding of the underlying mechanisms at the molecular scale [134,135,136,137,138,139,140,141,142,143,144,145,146,147,148,149]. However, MD simulations are computationally expensive and limited in terms of system size and simulation time [150,151,152,153]. Therefore, they are frequently paired with other simulation techniques and experimental data [119,120,154,155]. With some recent advances in this field such as coupling MD with machine learning and artificial enhancement approaches [156,157,158,159,160], metadynamics development [161], stochastic resetting algorithms [162], MD simulations could be sped up by several orders and become an emerging in silico technique to study the FSI phenomena in the near future.

### 3.4. Lattice Boltzmann Method

Lattice Boltzmann methods (LBMs) have emerged as a powerful numerical technique for studying fluid–structure interactions in microfluidics. LBMs describe the statistical behaviour of the particles in a fluid [163]. These methods discretise space and time into a lattice and simulate the movement and interactions of particles on the lattice. One advantage of LBMs is their ability to handle complex geometries and boundary conditions with parallel computing. LBMs have been widely used to simulate complex systems such as multiphasic fluids [164,165,166] and biological flows [167,168,169], and to study fluid–structure interactions [170,171,172,173] as well as rheological properties of red blood cells [174]. However, their application in micro elastofluidics remains unexplored, especially for FSIs.

Figure 8D illustrates the common lattice structures used in LBM simulations, namely the D2Q9 (two-dimensional with 9 velocities) square, D3Q19 (three-dimensional with 19 velocities), and D3Q27 (three-dimensional with 27 velocity vectors) cube lattice configurations. These structures are frequently employed in LBM simulations [175]. We use the D2Q9 model as an illustration to simplify the discussion of LBMs. Similar information regarding other models is readily accessible in the existing literature.

While considering the D2Q9 model, the following are the nine velocity vectors of lattice points [175].

(18) $$c_{i}=\left\{\begin{array}{c} 0\;\;\;\;\;\;\;\;\;\;\;\;\;\;\;\;\;\;\;\;\;\;\;\;\;\;\;\;\;\;\;\;\;\;\;\;\;\;\\ \left(\cos\frac{i-1}{2}\pi ,\sin\frac{i-1}{2}\pi \right)\frac{∆x}{∆t}\;\;\;\;\;\;\;\;\;\;\;\;,\;\;i=1-4\\ \sqrt{2}\left(\cos\frac{2i-9}{4}\pi ,\sin\frac{2i-1}{4}\pi \right)\frac{∆x}{∆t}\;\;,\;\;i=5-8 \end{array}\right.$$

The main variable in an LBM represents the fraction of particles travelling with a lattice velocity, within lattice site x and time t. For instance, $f_{0}$ represents particles at rest as $c_{0}=\left(0,0\right)$. Following a time step ∆t, $f_{i}\left(x,t\right)$ moves to an adjacent lattice site $x+c_{0}∆t$ along the lattice velocity $c_{i}$, a process known as streaming or propagation.

At that site, collisions occur among particles moving from different directions, altering the original particle numbers in each direction. As an outcome, another propagation system is started when a fresh set of density distributions with different lattice velocities emerge from the collision site. This sequence of propagation and collision processes recurs iteratively in LBM simulations until a satisfactory result is obtained.

The above dynamic process is mathematically formalised through the lattice Boltzmann equation (LBE) as [107]:

(19) $$f_{i}\left(x+c_{i}∆t,t+∆t\right)-f_{i}\left(x,t\right)=\Omega_{i}(f)$$

In this context, the collision operator Ω is in charge of figuring out distribution changes when the collision occurs. The propagation process previously explained is represented on the left-hand side of the equation using the Bhathagar–Gross–Krook (BGK) single-time approximation to approximate the collision operator, which is an important simplification of the LBM. Initially introduced for the Boltzmann equation within the continuum kinetic theory by Bhatnagar et al. [176], the lattice BGK (LBGK) equation can thus be expressed as:

(20) $$f_{i}\left(x+c_{i}∆t,t+∆t\right)-f_{i}\left(x,t\right)=-\frac{f_{i}\left(x,t\right)-{f_{i}}^{eq}(x,t)}{\tau }$$

where collision operator Ωᵢ is defined as:

(21) $$\Omega ᵢ=-\frac{{f_{i}}^{neq}}{\tau }$$

where τ is the BGK single relaxation time, and ${f_{i}}^{neq}$ is a non-equilibrium population that can be expressed as:

(22) $${f_{i}}^{neq}=f_{i}\left(x,t\right)-{f_{i}}^{eq}\left(x,t\right),$$

where ${f_{i}}^{eq}(x,t)$ is the discretised population at an equilibrium state at any point x and time t, which can be calculated by the discretisation of the Maxwell–Boltzmann equilibrium distribution [177] by the following polynomial:

(23) $${f_{i}}^{eq}=\rho \omega_{i}\left[1+\frac{u.c_{i}}{{c_{s}}^{2}}+\frac{1}{2}{\left(\frac{u.c_{i}}{{c_{s}}^{2}}\right)}^{2}-\frac{u.u}{{{2c}_{s}}^{2}}\right]$$

From the density distribution across the lattice, fluid density ρ and velocity u can be calculated as:

(24) $$\rho =\sum_{i}f_{i}$$

and

(25) $$\rho u=\sum_{i}f_{i}c_{i}$$

Here, $c_{s}$ is the lattice speed of sound, and $\omega_{i}$ is the lattice weight factor that depends on the lattice structure. For the D2Q9 model, $\omega_{0}=4/9$, $\omega_{1-4}=1/9$, $\omega_{5-8}=1/36$, and ${c_{s}}^{2}=∆x^{2}/{3∆t}^{2}$. By the Chapman–Enskog expansion, continuum macroscopic properties and momentum equation (Navier–Stokes Equation (2)) can be obtained from the above LBM discretised dynamics [178].

In the BGK single relaxation time, the kinematic viscosity υ and dynamic viscosity μ can be calculated as:

(26) $$\upsilon =(\tau -\frac{1}{2}){c_{s}}^{2}∆t$$

and

(27) $$\mu ={c_{s}}^{2}\rho (\tau -\frac{∆t}{2})$$

#### 3.4.1. Force Application in FSIs

Many microfluidic systems are subject to internal or external forces, including gravity [179], electric or magnetic forces [180,181], centrifugal force [182], and fluid–particle interactions [183]. The impact of a body force is conceptualised physically as the addition of momentum to the field. Therefore, a force component post-collision [184] is typically included in the LBE to account for this forcing influence on fluid dynamics. External forces are usually incorporated in the LB algorithm as a source term $S_{i}$ and a change to the equilibrium velocity $u^{eq}$ as follows [185]:

(28) $$f_{i}\left(x+c_{i}∆t,t+∆t\right)-f_{i}\left(x,t\right)=\Omega_{i}∆t+S_{i}∆t$$

and

(29) $$f_{i}\left(x+c_{i}∆t,t+∆t\right)-f_{i}\left(x,t\right)=-\frac{f_{i}\left(x,t\right)-{f_{i}}^{eq}(x,t)}{\tau }+S_{i}∆t$$

The expression of $S_{i}$ and $u^{eq}$ are not the same for all types of forcing schemes, for a physical force density F, multiple forcing schemes exist. This paper does not seek to cover all available forcing approaches. Instead, we highlight here the two prevalent forcing approaches: Guo’s [186] and Shan-Chen’s [187].

In the Guo scheme, the force source term $S_{i}$ is expressed as:

(30) $$S_{i}=\omega_{i}\left(1-\frac{∆t}{2\tau }\right)\left(\frac{c_{i}-u}{{c_{s}}^{2}}+\frac{u.c_{i}}{{c_{s}}^{4}}c_{i}\right).F$$

and the equilibrium velocity becomes:

(31) $$u^{eq}=\frac{1}{\rho }\sum_{i}f_{i}c_{i}+\frac{F∆t}{2\rho }$$

In the Shan-Chen scheme, the source term is zero, $S_{i}=0$, but the equilibrium velocity changes, including the effect of the external force:

(32) $$u^{eq}=\frac{1}{\rho }\sum_{i}f_{i}c_{i}+\frac{\tau F}{\rho }$$

These adjustments exhibit an increase in fluid momentum at a lattice node by F∆t per time step while preserving the fluid density. The above schemes can be used in simulating fully coupled fluid–structure interactions or one-way fluid–structure interactions by applying an external force through the lattice Boltzmann method [186,188,189,190].

#### 3.4.2. Boundary Conditions

In the LBM, substantial efforts are required to devise accurate and efficient boundary effects [191,192,193]. Most of the time, periodic boundary conditions are necessary at the inlet and outlet for simulating the fluid dynamics of repetitive or continuous systems. The periodic boundary conditions ensure consistent flow and properties by mimicking an infinite system. These conditions are particularly useful in reducing computational complexity and avoiding boundary-induced errors, making them vital for accurate and stable simulations.

In micro elastofluidics, where the flexible nature of materials poses unique challenges, wall–fluid boundary conditions are critical for accurately modelling fluid dynamics. Techniques such as simple bounce-back (SBB) [194] and mid-grid bounce-back [195] are used for rigid structures, while the modified bounce-back [194] and Immersed Body Method [196] are applied to flexible channels and deformable particles to effectively simulate flow interactions at boundaries. In SBB, the particle simply reflecting, bounces backward to the initial node $x_{f}$ in the opposite direction. The after-collision population ${f_{i}}^{*}$ moving from a fluid node $x_{f}$ to a solid node $x_{s}$ in the SBB method is shown in Figure 8E(i). The bounce-back population both in the bounce-back method or mid-grid bounce-back method can be expressed as [195]:

(33) $$f_{\bar{i}}\left(x_{f},t+∆t\right)={f_{i}}^{*}\left(x_{f},t\right)$$

In this approach, the no-slip condition can achieve second-order accuracy when the physical wall is straight, parallel with one of the main lattice axes, and positioned midway between $x_{f}$ and $x_{s}$.

For managing curved boundaries, the interpolated/extrapolated bounce-back method is the most popular enhancement that is applicable to the SBB technique [197,198]. This method considers the separation between the actual wall position and the lattice nodes, as shown in Figure 8E(ii). In this case, following the collision step, a hypothetical distribution is presumed to depart from the solid node $x_{s}$ and traverse toward the fluid node $x_{f}$ along a lattice direction and approximated as [199]:

(34) $${f_{\bar{i}}}^{*}\left(x_{s}\right)={\left(1-X\right)f_{i}}^{*}\left(x_{f}\right)+X{f_{i}}^{*}(x_{b})$$

where the two terms on the right-hand side are the interpolation between $x_{f}$ and $x_{b}$ with X the contributing factor as defined by Filippova et al. [199].

In micro elastofluidics, addressing moving boundary conditions is important when dealing with dynamic interfaces or deformable structures. As discussed before, methods like modified bounce-back and immersed boundary are used for modelling the dynamic interactions between fluids and deformable structures. In the former method, the bounce-back technique is modified to consider scenarios involving moving boundaries, whether they are due to externally imposed motion or the effect of a fully coupled fluid–structure interaction. The momentum transfer at a moving boundary is captured by incorporating a correction term in the SBB technique, introduced by Ladd et al. [194] as:

(35) $$f_{\bar{i}}\left(x_{f},t+∆t\right)={f_{i}}^{*}\left(x_{f},t\right)-\frac{2\omega_{i}\rho_{w}c_{i}u_{w}}{{c_{s}}^{2}}$$

where $u_{w}$ and $\rho_{w}$ are the wall velocity and density, respectively. For the Interpolated Bounce-Back (IBB) method, the correction term needs to be implemented on the segment of the population after the collision that moves into the wall boundary [200].

### 3.5. Immersed Body Method in FSIs

Originally intended for deformable membranes inside a flow field, the immersed boundary method (IBM) was created by Peskin [196]. An integral relationship can be used to calculate the membrane force from membrane deformation. Membrane forces are transferred to the local fluid as part of fluid-membrane interactions and the membrane configuration was updated in response to the local flow velocity. This method has the advantage of avoiding problems related to shifting boundaries by allowing numerical techniques to solve fluid flow on a fixed, regular Eulerian mesh. In microfluidics, the immersed body approach has been effectively combined with a solver for both soft and rigid particles [201,202,203,204]. This technique has also been used to investigate the dynamics of RBCs in microcirculation [205,206].

When a fundamental relationship of solid boundaries is not available, alternative relationships must be developed between the desired boundary velocity and the boundary force to stimulate solid particles and moving boundaries in a flow. Many approaches have been proposed. For example, Feng et al. [173] proposed modelling solid particles as deformable, using a spring force to represent interactions between each particle and a virtual reference point. This approach allowed the simulation of how particles elastically respond to displacement, aiding in understanding their behaviour under various stress conditions. Niu et al. [207] determined the boundary force through the momentum interchange of particle distributions at the boundary. Dupuis et al. [208] used the LBM with the IBM for modelling the Navier–Stokes Equation (2). The boundary force was calculated by comparing the desired boundary velocity with that computed without the boundary force to account for the no-slip boundary condition.

The IBM uses the Lagrangian description for the immersed structures and the Eulerian description for the fluid flow. This method takes into account the movement and distortion of immersed bodies while accurately representing fluid behaviour. In order to take into consideration the impact of immersed structures on surrounding fluids, this approach utilises forcing functions. These features allow forces to be transferred from the fluid to the immersed bodies, enabling the fluid to act on the structures and vice versa. A collection of Lagrangian points or markers positioned within the Eulerian grid serves as the representation of immersed boundaries. Interpolation techniques compute the influence of these markers on the surrounding fluid grid as shown in Figure 8F.

Methods such as interpolation and projection are employed to transfer information between the Eulerian and Lagrangian domains, ensuring the accurate exchange of forces, velocities, and displacements between the fluid and structures. The typical IBM process involves several key steps.

The first step is interpolating the velocity of fluid u(X) at position $x_{i}$ of every particle mesh vertex i in Figure 8F. The interpolated velocity ${\dot{x}}_{i}$ is given by [87]:

(36) $${\dot{x}}_{i}={∆x}^{d}\sum_{X}u(X)\delta (X-x_{i})$$

where d is the number of spatial dimensions, ∆x is the lattice spacing, δ and is a discrete delta distribution. After interpolating the fluid velocity, the force $f_{i}$ acting on each vertex i is calculated. For soft particles, the forces resulting from the mesh deformation caused by moving vertices lead to the changing distance $\left|x_{i}-x_{j}\right|$ over time. Then, each vertex force $f_{i}$ is spread over the fluid, treating them as body forces F(X) according to [87]:

(37) $$F\left(X\right)=\sum_{i}f_{i}\delta \left(X-x_{i}\right)$$

After spreading the force as per Equation (37), the method updates the position and orientation of each particle based on the forces acting on it. The position of the vertex can be updated as:

(38) $$x_{i}\left(t+∆t\right)=x_{i}\left(t\right)+\dot{x_{i}}\left(t\right)∆t$$

The shape of the discrete delta distribution $\delta \left(X-x_{i}\right)$ is crucial, and a common simplification is to use factorised 2D or 3D kernel functions.

For soft particles, the vertex velocity is determined by interpolating the fluid velocity. Particle deformation is caused by moving vertices. For rigid particles, challenges involve satisfying rigidity conditions and no-slip conditions simultaneously. Various IBM algorithms for rigid particles exist, such as direct-forcing, implicit IB, and multi-direct-forcing methods.

When a system features symmetric or periodic spatial characteristics, symmetric or periodic boundary conditions can be effectively applied to reduce the computational domain and enhance efficiency. These conditions are particularly well suited to simulations using the IBM due to their adaptability with particle-based models. For periodic boundaries, particles that exit the domain on one side are reintroduced from the opposite side, effectively simulating an infinitely extended horizontal domain with identical repeating units. Additionally, to implement a pressure gradient in a channel, the periodic boundary condition can be modified to account for a pressure (density) variation between the domain’s inlet and outlet [193].

The two main approaches for flow in geometries with streamwise periodic boundary conditions are (i) imposing a pressure drop ∆p on the periodic boundary condition and (ii) a body force F. The previous approach, which can be substituted by a constant body force $\left|F\right|=∆p$, works well in straight channels with an effective constant pressure gradient. It is often preferable to apply an overall pressure drop ∆p between the inlet and outlet for other geometries. Then, taking into consideration any pressure variation brought on the particles, the LB algorithm adjusts for the proper pressure field inside the domain. Periodic boundaries are conceptually simple, but they have some difficulties as well. Controlling long-range particle–particle interactions across boundaries is necessary to simulate an infinite number of particles. This frequently involves sensitivity tests of selected channel length [209,210].

Creating more appropriate boundary conditions for complex geometries remains a difficult task. Distortion in the flow field can spread downstream in inertial flows. Any upstream influence is ignored when using a fully formed velocity profile at the inlet, which is not suitable for obtaining accurate results. Further research is required to facilitate practical and accurate LB simulations of microfluidic devices that cannot be approximated with periodically repeating boundary conditions.

Choosing the right method depends on the specific requirements of the simulation scenario. These computational methods and their specific attributes are summarized in the Table 1, providing a comprehensive comparison to aid in the selection of the most appropriate method based on the diverse needs of different simulation scenarios.

## 4. Applications

Computational FSI methods are pivotal in micro elastofluidics, enhancing the design and functionality of devices such as microvalves, micropumps, and micromixers. These techniques enable the precise modelling of fluid and structural dynamics, critical for devices that control and manipulate fluid flow at the microscale. FSI methods are also instrumental in biomedical applications, including cell separation and particle manipulation. Additionally, in cardiovascular applications, FSI methods help to develop devices that match the biomechanical properties of blood and vascular tissues, significantly improving the intended therapeutic purpose. This underscores the integral role of FSI in advancing micro-elastofluidic technology across various scientific and medical fields.

### 4.1. Microvalves and Micropumps

Microvalves and micropumps are the typical microfluidic components with strong FSIs. The FSI dictates how flexible membranes and channels within these devices respond to fluid pressure, ultimately shaping their ability to regulate and deliver tiny volumes of liquid in lab-on-a-chip and drug-delivery devices. Much work has been conducted on analysing the intricate interplay between fluid forces and deformations of flexible structures. Researchers have proposed designs of microvalves and micropumps that achieve unparalleled precision in flow control.

Among the different types of microvalves, elastomeric membrane [211] microvalves are a prime example of where FSI plays a critical role [212]. The core principle behind these valves is the deformation of a flexible membrane in response to fluid pressure [213]. Active valves often utilise external actuation through FSI for precise flow control. Various actuation mechanisms have been proposed [214,215,216,217,218]. Passive valves harness flow forces through FSI to achieve remarkable self-regulation [219,220,221]. In a passive microvalve, the fluid pressure deforms the membrane, which in turn alters the flow resistance. This dynamic interplay between the fluid and the membrane allows the valve to maintain a constant flow rate over a specific pressure range.

**Figure 9:**
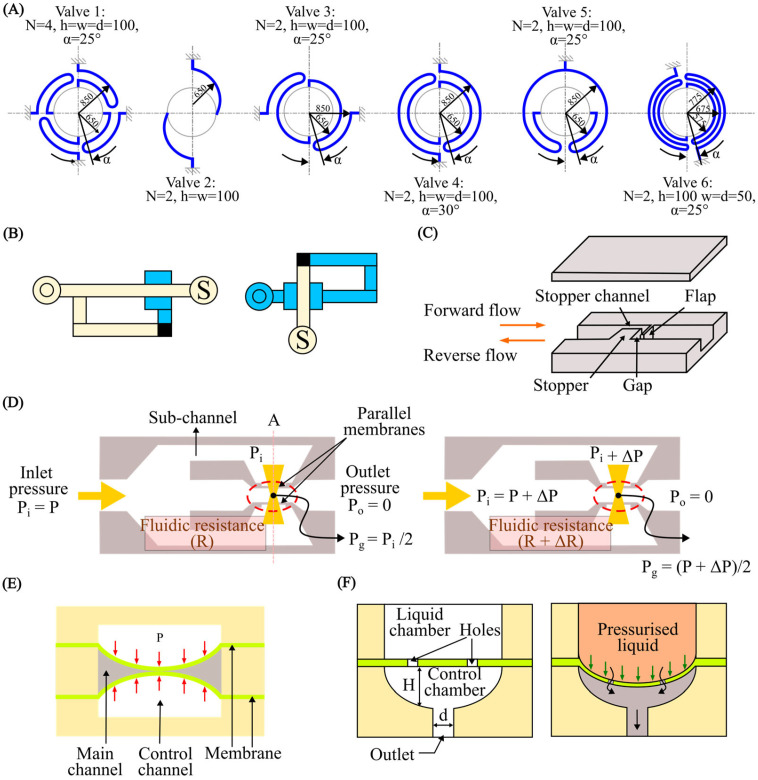
Microvalves and micropumps. (**A**) Ortho-planar micro check valves with different stiffness values [222]; (**B**) Poiseuille law pressure-drop self-regulating valve [223]; (**C**) self-adaptive planar check valve with flexible cantilever flap [224]; (**D**) parallel membrane with low threshold pressure, self-regulating valve [225]; (**E**) Stacked parallel membrane with low threshold pressure, regulating valve [226]; (**F**) ellipsoid control chamber auto-regulating valve [221].

Numerous models for passive check valves and passive regulating valves have been proposed. For instance, Nguyen et al. [222] utilised FSI to study passive valves and proposed models for ortho-planar micro check valves for incorporation in polymeric microdevices (Figure 9A). These check valves efficiently prevented backflow and require an inlet pressure of less than 1 kPa to open. Ortho-planar designs provided enhanced sealing performance, thanks to their parallel out-of-plane motion. Later, Kartalov et al. [223] proposed a PDMS push-up valve utilising the pressure drop along the channel length and performed an FSI simulation to investigate the flow rate and threshold pressure (Figure 9B). This model maintained a constant flow rate of 0.033 mL/min with a threshold pressure of 103 kPa. Yang et al. [224] designed a planar check valve and did an FSI study to model self-adaptive variable resistors to use in microfluidics models, Figure 9C. This valve design achieved a relatively high flow rate of 1.2 mL/min with a threshold pressure of 100 kPa. In addition, Doh et al. [225] developed a passive parallel membrane valve designed for low-threshold pressure operation using the concept of FSI (Figure 9D). The valve consisted of two control channels, two vertically oriented membranes, and a single fluidic channel. The autonomous deflection of the membranes within the microchannel enabled the valve to achieve flow regulation at a pressure as low as 15 kPa. Moreover, Zhang et al. [226] utilised FSI on flow regulation in microfluidic environments and developed a unique parallel membrane valve featuring a stacked five-layer architecture (Figure 9E). This design, with two horizontal membranes enclosing a fluidic channel and sandwiched between control channels, achieved a remarkable flow rate of 2.79 mL/min with a low 10 kPa threshold pressure. Zhang et al. [221] achieved low threshold pressure in microfluidic high-throughput delivery systems and designed a passive valve for stable flow control. The valve utilised an ellipsoid control chamber and a dual micro-hole elastic membrane (Figure 9F). Membrane deflection in response to the pressurised flow through the micro-holes dynamically modified the control chamber’s resistance. This self-regulating mechanism maintained a constant flow rate regardless of inlet pressure changes.

**Figure 10:**
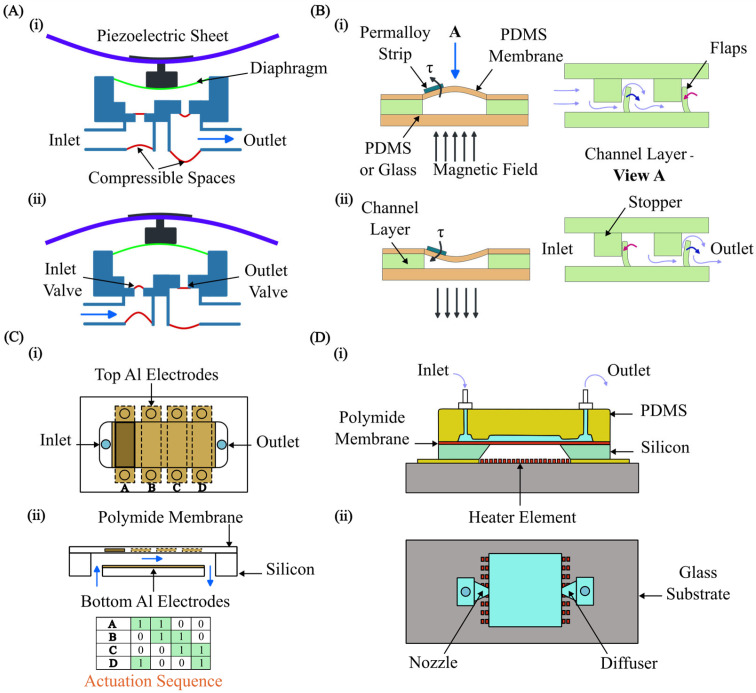
Pumping schemes. (**A**) Piezoelectric micropump utilising fixed-end PDMS valves with integrated compressible space: (**i**) dispensing mode, (**ii**) absorbing mode [232]; (**B**) magnetically actuated membrane micropump with in-plane check valves: (**i**) priming mode, (**ii**) pumping mode [233]; (**C**) electrostatically actuated micropump utilising four electrodes to induce peristaltic motion: (**i**) top-view, (**ii**) cross-sectional view [234]; (**D**) thermo-pneumatic micropump with a thin polyimide membrane actuator: (**i**) cross-sectional view, (**ii**) top view [235].

A micropump is another crucial component of many microfluidic systems. Since their invention, micropumps have seen significant advancements, offering advantages such as compact size, portability, energy efficiency, a wide range of flow rates, affordability, and potential for integration with other microfluidic components. Micropumps are typically constructed using micro-electromechanical systems (MEMS) techniques on biocompatible materials such as silicon, glass, or various polymers (such as polymethyl methacrylate (PMMA), PDMS, or SU-8 photoresist) [227,228]. Micropumps fall into two broad categories: (i) mechanical, using moving parts like diaphragms and valves, and (ii) non-mechanical, which manipulates fluid flow through hydrodynamic [229], electroosmotic [230], or electrowetting [231] forces. In mechanical micropumps, fluid flow is pressurised by an external force, i.e., piezoelectric (PZT), electromagnetic (EM), electrostatic, and thermo-pneumatic, applied to either fixed flexible membranes or moving structures. This force transfer to the fluid occurs through fluid–structure interaction. Much work has been done in harnessing and manipulating the pumping function at the microscale through FSI. Wang et al. [232] proposed a piezoelectric micropump utilising fixed-end PDMS valves with integrated compressible space. This micropump utilised a resonantly driven membrane actuator, two fixed-end PDMS check valves for stability and reduced leakage, and strategically placed compressible spaces (Figure 10A).

A piezoelectric actuator deforms the pump membrane. This deformation of the membrane affects the fluid inside the pump by changing the space it occupies, which increases or decreases the pressure. Essentially, as the membrane changes shape, it pushes on the fluid, helping to move it through the system. The micropump design relies on two key interactions: (i) the electromechanical interaction, where the piezoelectric sheet converts electric signals into a movement of the beam, and (ii) the fluid–solid interaction, where the pump diaphragm interacts with the working fluid. An alternating voltage causes the beam to deform, driving the membrane and, thus, the fluid flow. Simultaneously, the fluid resists the movement of the membrane. The reported micropump delivers a maximum flow rate of 105 mL/min and a maximum back pressure of 23 kPa under a 400 V sinusoidal voltage at 490 Hz. The maximum power consumption at zero back pressure is approximately 42 mW.

Ni et al. [233] introduced a magnetic micropump utilising FSI for easy fabrication and seamless integration into other microfluidic systems. The device featured in-plane check valves for flow control and a magnetically actuated membrane (Figure 10B). The deformable elastic membrane then interacted with the flowing fluid and, in turn, pressurised the fluid. Since actuation was controlled directly by an external magnetic field, enabling efficient wireless operation, this method was ideal for various applications. Experimental results indicated that the micropump could deliver 0.15 μL/min at 2 Hz, offering 1 nL per stroke resolution, and worked against 550 Pa back pressure.

Moreover, Lee et al. [234] fabricated an electrostatically actuated micropump incorporating FSI, utilising four electrodes to induce peristaltic motion (Figure 10C). In this study, the micropump made use of the electrostatic force to create bidirectional peristaltic motion. Its unique design featured a single deformable membrane with four movable polyimide electrodes hence eliminating the need for valves. Actuation signals caused the membrane to bend in small, sequential steps, which increased the pressure of the fluid in stages. This action splits a single chamber into two, three, or four separate sections, allowing for the controlled movement of the fluid within the device. Experiments showed that optimising the actuation signal dramatically increased the flow rate. With a basic signal, the pump achieved 38 μL/min, but an optimised signal boosted this to 136 μL/min (both at 90 V and 15 Hz). This represented a 3.6-fold improvement. Hamid et al. [235] modelled a cost-effective thermo-pneumatic micropump with a thin polyimide membrane actuator (Figure 10D). The model included a microheater, thermal cavity, and planar valve. This thermo-pneumatic micropump utilised thermal air expansion within a chamber to actuate a thin polyimide membrane. The membrane movement then interacted with the fluid and hence created pressure fluctuations accordingly. This device effectively controlled fluid on the picolitre to nanolitre scale, making it suitable for applications such as artificial kidneys and drug delivery systems, and was both simple and economical to fabricate.

### 4.2. Cell and Particle Manipulation

Cell sorting is a laboratory technique for isolating a specific cell type from a mixed population. Isolation criteria include physical parameters (size, morphology), cell viability, and the presence of specific intracellular or extracellular proteins [236]. Purified cells obtained through sorting are essential tools for research, diagnostic procedures, and cell-based therapies. Cell sorting encompasses a broad range of established techniques, employing both active and passive mechanisms [237]. Active sorting utilises external fields (electric, acoustic, magnetic, or optical) to manipulate cell trajectories. Passive systems primarily leverage inertial forces, filtration, and cell-surface adhesion for purification. Understanding FSIs in both cell–fluid and fluid–channel interactions is crucial for optimising and designing cell sorting devices. Extensive experimental works have been conducted to utilise FSI phenomena for cell sorting and manipulation. However, numerical simulations are vital to fully comprehend these FSI phenomena. Numerical modelling of cell dynamics complements experimental approaches, enabling the in-depth study of fluid–particle interactions. Accurately simulating these dynamic processes presents challenges due to the complexity of FSI coupling, cell mechanics, and the computational cost of simulating cell–cell interactions at scale.

**Figure 11:**
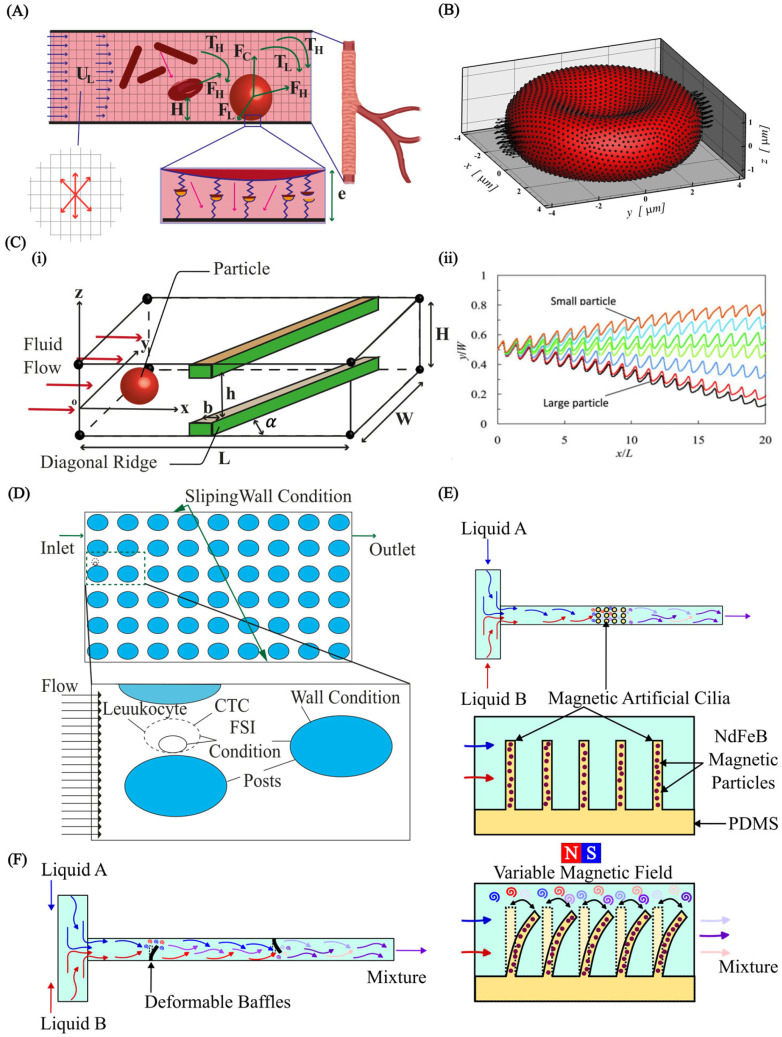
Cell separation and micromixers. (**A**) Illustration of considering RBCs and WBCs as suspended particles in plasma to capture fluid–particle and fluid–wall interactions [238]; (**B**) RBC discretisation to investigate the change in deformability of cells caused by malaria parasites, reproduced with permission from Hosseini et al. [240]; (**C**) (**i**) setup for particle sorting in microchannels, (**ii**) particle separation, reproduced with permission from Mao et al. [239]; (**D**) DLD device for separating CTCs within blood stream [241]; (**E**) mixing with magnetic actuated artificial cilia [242]; (**F**) passive mixing with flexible baffles [243].

Sun et al. [238] developed an LBM model to simulate blood flow in realistic microvascular networks and the separation of different types of cells (Figure 11A). This approach allowed for the detailed analysis of the FSI between blood cells and the vessel wall. This model treated blood as a suspension of particles (red blood cells [RBCs] and white blood cells [WBCs]) within the plasma, explicitly incorporating cell–cell and cell–wall interactions. The LBM approach allowed the simulation of RBC and WBC interactions as the cells flowed through a microvascular network. This approach enabled (i) the quantification of forces exerted between RBCs and WBCs, the tracking of trajectories of individual cells, (ii) the analysis of pressure variations within the network due to cellular traffic, and (iii) the evaluation of forces experienced by the vessel walls at any location. Simulations demonstrated that the vessel curvature and junctions increased the apparent viscosity and induced stress perturbations near stagnation points. The results suggested a potential link to atherogenesis at stagnation points and may also significantly influence our understanding of endothelial biology and its role in atherosclerosis formation.

Mao et al. [239] conducted computational modelling of particle sorting, specifically addressing FSI, for high-throughput hydrodynamic size-based sorting of solid microparticles in microchannels (Figure 11C(i)). With this model, high-resolution separation was achieved by combining the cross-stream inertial migration of particles with circulatory flows induced by periodic diagonal ridges on opposite channel walls. A hybrid approach was employed to model the multi-component system of a fluid-filled ridged microchannel and various-sized solid particles. This approach integrated the LBM for fluid dynamics with a lattice spring model (LSM) for modelling solids. The FSI was captured through appropriate boundary conditions at the solid–fluid interface. Simulations proved to be crucial for designing the ridged microchannel. Optimisation for separating neutrally buoyant microparticles by size relied heavily on understanding the complex FSI within a microchannel. Figure 11C(ii) shows the results of this study. The geometry of the microchannel induced unique fluid flow patterns, and the resulting FSI between these patterns and the particles was the key to high-resolution separation.

Khodaee et al. [241] carried out a numerical FSI study of the fluid–particle interaction in a deterministic lateral displacement (DLD) microfluidic device for the effective separation of circulating tumour cells (CTC) in bloodstreams. Numerical simulations, incorporating FSIs using the FEM to model deformable cell behaviour, guided the design of a typical DLD array for separating CTCs and leukocytes (white blood cells) under various flow conditions. Figure 11D illustrates the discretisation of this method. This study focused on how coupled FSI phenomena, specifically related to flow conditions, stress, and cell deformability impacted cell separation in DLD devices. This model provided essential data for optimising DLD devices, enhancing efficiency, and protecting cell viability. The model quantified the cellular stress experienced during separation and mapped the distribution of effective stress at peak deformation. Moreover, Gul et al. [244] presented a computational model using a two-way coupled bio-magnetic fluid dynamics approach to simulate targeted magnetic drug delivery (TMDD), leveraging magnetic nanoparticles (MNs) coated with anticancer drugs for optimised navigation within blood vessels. The model successfully incorporated active magnetic particle manipulation, enhancing the precision and efficacy of drug delivery to targeted areas.

FSI also plays a fundamental role in accurately simulating cell deformations within a complex environment. Cells are not rigid bodies and respond dynamically to the fluid forces surrounding them. These forces can cause cells to stretch, compress, and change shape. In turn, deformed cells alter the flow field around them. FSI models are essential for capturing this intricate interplay. An FSI simulation provides realistic predictions of how cells deform under various conditions. This has far-reaching implications in biomedical research, understanding disease processes where cell deformation plays a role. For instance, Hosseini et al. [240] carried out an FSI analysis on RBCs and investigated the change in the deformability of cells caused by malaria parasites. Numerical simulations incorporating FSIs were employed to investigate this phenomenon. The cell membrane was represented as a collection of interconnected, elastic particles (Figure 11B). The cytosol was modelled as a Newtonian fluid using smoothed particle hydrodynamic techniques (SPH). More importantly, the malaria parasite was treated as a rigid structure, capturing its influence on the overall behaviour of the cells. Healthy RBCs are remarkably flexible, but the presence of the rigid malaria parasite within the cell significantly increases their stiffness. In response to the fluid forces present in the bloodstream, the deformability of infected cells declines. These findings underscore the potential of using cell deformability as a diagnostic marker and highlight the value of FSI simulation in understanding disease-induced cellular changes.

### 4.3. Micromixers

Mixing fluids at the microscale presents unique challenges and opportunities. Due to the dominance of laminar flow and the lack of turbulence at the microscale, traditional mixing strategies often become ineffective. This challenge has spurred the development of innovative microfluidic mixing approaches, relying on strategies such as chaotic advection, diffusion enhancement, or the integration of active micromixers. Micromixers are broadly categorised as active and passive types [245]. Active micromixers enhance mixing by introducing external perturbations that disrupt the typically laminar flow regime. Methods include pressure-driven actuation (e.g., pulsatile flows), electrokinetic manipulation (e.g., electroosmotic flow), magnetic actuation (e.g., ferrofluid mixing), or acoustic streaming [246,247,248,249,250,251,252]. These techniques offer rapid mixing, tunability, and adaptability but come with increasing system complexity. In contrast, passive micromixers rely on the microchannel geometry to promote mixing. Complex channel geometries induce chaotic advection, increasing interfacial contact area through lamination, splitting, or droplets [253,254,255,256,257,258]. Passive mixers excel in simplicity, cost-effectiveness, and minimal sample perturbation, but may have longer mixing times and less adaptability than their active counterparts.

FSI plays a vital role in both active and passive micromixers. In active micromixers based on FSI, flexible elements interact with the fluid flow, creating complex patterns that disrupt the laminar flow for fast and efficient mixing. This FSI approach allows for customised flow control, works with various fluids, and potentially uses less power than other active mixing methods. Examples include employing deformable membranes to create chaotic advection or integrating oscillating microstructures to induce localised mixing zones. While less common than active FSI micromixers, passive designs can also exploit FSI to enhance mixing. These mixers often incorporate flexible or deformable elements within the microchannel that respond to the inherent fluid forces. Examples include flexible micro-posts that sway in response to flow, membranes that deform under pressure, and integrated microvalves whose operation is triggered by fluid forces.

Much work has been conducted on optimising the design of micromixers using FSI-based numerical simulation. For example, Lin et al. [242] carried out an FSI analysis for precise flow manipulation in a micromixer using magnetic actuation. The team employed microstructures with embedded magnetic particles (Figure 11E). A CFD approach utilising FSI modelling was employed to simulate the flow patterns generated by the actuated structures. The model revealed the impact of different actuation modes on mixing performance. The “zigzag” pattern proved to be superior in achieving rapid and complete mixing. A further analysis demonstrated how these structures disrupted and blended the flow, with vorticity calculations pinpointing regions of high vorticity in the flow field. The enhanced vorticity strongly correlated with improved mixing. The study featured the power of combining experimental and numerical analyses to understand the FSI mechanisms responsible for effective flow mixing. These findings hold significant value for designing future high-performance micromixers, where speed and thorough mixing are essential. Moreover, Talebjedi et al. [243] exploited the FSI phenomenon in passive micromixers using flexible baffles (Figure 11F). This research explored the use of deformable baffles in the mixing process, aiming to improve performance compared to traditional rigid baffles. Modelling the FSI provided insight into how deformable baffles changed shape under fluid pressure, and how this affected flow. The results showed a significant reduction in pressure drop with deformable baffles, indicating less stress on mixed materials. More importantly, this improvement was achieved with only a minor decrease in mixing efficiency as compared to rigid baffles. This suggests that deformable baffles offer a promising way to optimise mixing processes, where reducing stress on the materials being mixed is vital.

### 4.4. Modelling Cardiovascular Systems

In the realm of micro elastofluidics, computational methods for FSI unlock a deeper understanding of the complex interplay between biological fluids and the flexible tissues they encounter. The complex dynamics of blood flow, coupled with the flexible nature of heart valves and arterial walls, necessitate FSI simulations for a comprehensive analysis of the cardiovascular system. FSI models provide critical insights into heart valve function, including leaflet deformation, flow patterns, and stress distribution, leading to better diagnostics for heart diseases and improved designs for prosthetic replacements [259,260,261,262,263]. Similarly, FSI models applied to artery flow provide insights into the development of arterial diseases like atherosclerosis, uncovering potential risks for aneurysm formation, and contribute to optimising medical devices such as stents [46,264,265,266,267].

**Figure 12:**
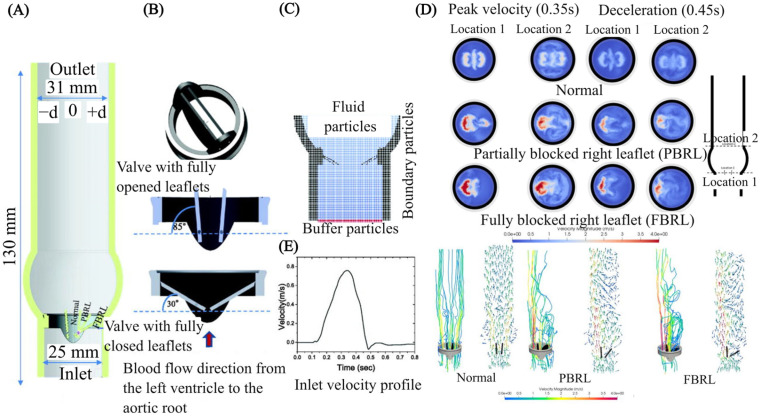
Bi-leaflet mechanical heart valve dynamics through FSI modelling with Smoothed Particle Hydrodynamics (SPH); (**A**) illustration of mechanical heart valve; (**B**) opening and closing position of valve; (**C**) illustration of smoothed particles for simulation; (**D**) inlet velocity profile to mimic the real pulse; (**E**) simulation results. Reproduced with permission from Laha et al. [259].

Heart valves play a critical role in maintaining blood flow direction. Artificial valves are treatment options, but their design and performance require careful analysis. Laha et al. [259] investigated bi-leaflet mechanical heart valve dynamics through FSI modelling with Smoothed Particle Hydrodynamics (SPH). Figure 12 illustrates the schematic model. The team explored a method for simulating a bi-leaflet heart valve using SPH open-source code. By incorporating the FSI, the SPH technique analysed hemodynamic abnormalities associated with valve dysfunction. The study considered normal and abnormal flow behaviour, valve movement under blockage scenarios, and potential risks associated with blockages. The findings demonstrated the effectiveness of this SPH/FSI approach for capturing the dynamic behaviour of bi-leaflet valves. The versatility of this computational model suggests its potential application to more complex cardiovascular problems.

**Figure 13:**
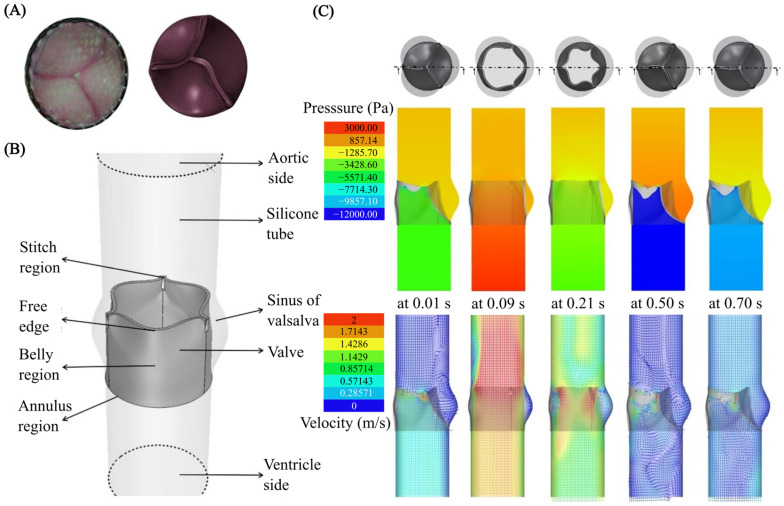
An artificial aortic heart valve. (**A**) Effect of asymmetry on valve closure (**right**) and comparison with a similar tex-valve (**left**); (**B**) illustration of test setup for FSI simulations; (**C**) simulation results. Reproduced with permission from Sodhani et al. [260].

Sodhani et al. [260] carried out an FSI study on an artificial aortic heart valve that was reinforced with textiles. In this study, an in silico FSI model was developed using the immersed boundary method to mimic the in vitro experiment. The model assessed the geometric orifice area and flow rate over a single cycle while also incorporating the material properties of the implant. The model employed fixed boundary conditions for the structural part. This involved fixing the bottom and stitched regions of the device in all directions, preventing movement in those areas. For the fluid domain, the transvalvular pressure was incorporated as a boundary condition at the inlet and a zero-pressure condition at the outlet. The transvalvular pressure was measured in the corresponding in vitro test. Figure 13 shows the model of the heart valve. The FSI simulation provided valuable insights into device performance, i.e., pressure distribution, velocity field, recirculation zones, vortices, and potential leakage points. This work demonstrated the effectiveness of FSI simulations for validating material property determination techniques and predicting the kinematics and flow behaviours of the device.

**Figure 14:**
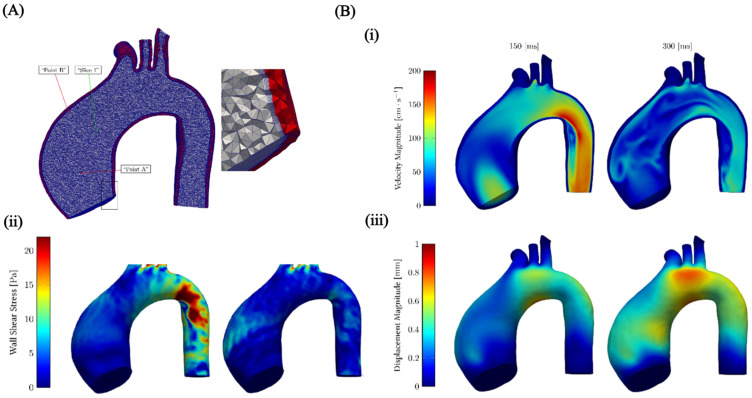
Numerical investigation of an Ascending Thoracic Aortic Aneurysm (ATAA) through FSI simulations. (**A**) Mesh discretisation of patient-specific geometric ascending thoracic aorta model reconstructed from CT scans; (**B**) simulations results. (**i**) wall shear stress distribution over the domain, (**ii**) Velocity magnitude distribution over the domain, (**iii**) Displacement magnitude distribution over the domain. Reproduced with permission from Valente et al. [268].

Arterial diseases, including atherosclerosis and aneurysms, pose significant health risks due to their potential to cause serious cardiovascular events such as heart attacks and strokes. By accurately simulating the dynamic interactions between blood flow and arterial walls, FSI models provide invaluable insights into mechanical forces that contribute to disease progression. For example, Valente et al. [268] carried out the numerical investigation of an Ascending Thoracic Aortic Aneurysm (ATAA) through FSI simulations using the open-source software package SimVascular. Figure 14A,B illustrate the mesh model and results, respectively. The simulations were based on patient-specific geometric models reconstructed from Computed Tomography (CT) scans. The analysis incorporated specific outlet conditions and temporal flow variations at the model inlet. By assigning prestress, the aorta model accurately reflected the in vivo stress state during the cardiac cycle. The process began with a CFD analysis on the fluid domain, followed by a structural analysis in the solid domain, using the pressures from the CFD phase as boundary conditions. The results from both CFD and Computational Structural Mechanics (CSM) were used as initial conditions for further analysis. The hemodynamic and structural behaviour of an ATAA was studied, focusing on the velocity, displacement magnitudes, and wall shear stress distribution during the first cardiac cycle. The results confirmed the effectiveness of the simulation in capturing the complex dynamics of an ATAA, highlighting its potential for enhanced precision in biomechanical assessments.

## 5. Perspectives

The exploration of FSIs in microfluidics using both continuum and particle computational approaches presents a dynamic and promising research area. Computational FSI and the deformable nature of structures in microfluidic devices have created new opportunities for applications across diverse fields, including biotechnology, healthcare, and soft robotics. However, the selection of an appropriate computational method depends heavily on the specific requirements and constraints of the application. Among the computational methods, the FEM is highly valued for its robust handling of complex geometries and diverse material properties, making it ideal for scenarios involving significant structural deformations. However, the computational cost makes the FEM less efficient for fluid dynamics problems as compared to other methods. The BEM offers an advantage for fluid flow around structures by reducing the problem dimensionality since it only requires discretisation at the boundaries. The dimension reduction leads to a substantial saving of computational cost. However, the BEM struggles with nonlinear properties and dynamically changing domains. In contrast, the LBM is celebrated for its simplicity and efficiency in handling fluid flows and multiphase phenomena, easily integrated with complex boundary conditions. However, coupling the LBM effectively with solid mechanics models remains challenging. MD provides exceptional detail at the molecular level, perfect for analysing microscale interactions within fluids and at fluid–solid interfaces. Yet, its practical application is typically limited to small systems due to the high computational demands. On the other hand, the IBM is particularly effective for problems where fluid and structure are dynamically interlinked, such as in situations with complex and moving boundaries, when structures within the fluid change position or shape. Despite its versatility, the IBM requires careful calibration to accurately capture the dynamics of the fluid–structure interface.

Looking ahead, the potential for computational FSI in micro elastofluidics is vast, with opportunities to combine the strengths of the above methods through hybrid modelling approaches. For instance, integrating the LBM’s fluid dynamics capabilities with the FEM’s structural dynamics expertise could yield an approach that leverages the strengths of both LBM and FEM, offering more accurate and comprehensive simulation. As computational resources continue to expand and algorithms evolve, the scope for real-time simulations and enhanced multiscale modelling will broaden, paving the way for more precise and efficient designs.

Real-time simulation capabilities will pave the way for adaptive microfluidic systems that dynamically respond to environmental changes, ideal for biomedical implants and wearable applications. The integration of Artificial Intelligence (AI) will streamline the design process, optimise simulation parameters, and predict system behaviours under diverse conditions, reducing development time and improving device efficacy. Moreover, the focus on sustainable technologies will encourage the integration of biodegradable materials into micro elastofluidic devices, necessitating robust FSI methods to assess their performance. Personalised medicine will also benefit from FSI-optimised devices tailored to individual physiological conditions, optimising diagnostic and therapeutic outcomes. Overall, these recent advances will significantly enhance the scope and effectiveness of micro elastofluidic applications, transforming both technological capabilities and their societal impact.

## Figures and Tables

**Table 1:** Summary of computational methods and applications.

Feature	FEM	BEM	MD	LBM	IBM
Significance	Uses discrete elements Solves field variables	Reduced dimensions Solves integral equations	Uses Newton’s laws of motion	Uses discrete particle distribution functions	Embeds the structure in a fluid mesh
Primary Applications	Structural analysis Micropumps Microvalves	Acoustic streaming Infinite domain flows	Biosensors Drug delivery	Droplet generation Multiphase flows	Immersed-structure FSI Cardiovascular simulations
Computational Domain	Discretises entire volumetric domain	Discretises boundary surfaces only	Simulates individual particles at molecular scale	Based on a fixed grid of discrete points	Combines fluid mesh with non-conforming structures
Strengths	Handles complex geometries/multiphysics	Minimum discretisation requirement Faster for small boundaries	Detailed molecular-level information	Efficient for complex boundary conditions Scalable for large systems	Handles FSI without mesh conformity
Weaknesses	Computationally intensive for large/complex domains	Limited to defined boundary problems	Computationally intensive Limitation in system size	Dependency on lattice resolution	Effects on accuracy near fluid–structure boundary
Mesh	Highly mesh-dependent The accuracy increases with finer mesh	Only the boundary needs meshing	No traditional mesh Particle density and interaction range are crucial	Mesh (lattice)-dependent Less sensitive than FEM	Mesh-dependent Fluid mesh should be fine enough for accuracy
Solvers	Direct Iterative	Direct Boundary integral	Verlet integration Velocity Verlet leapfrog	Collision and streaming operators	Direct forcing Lagrangian–Eulerian
Software	ANSYS (2024R1) Abaqus (2023) COMSOL (6.2)	ANSYS (2024 R1) BEASY (2024) Altair AcuSolve (2023)	LAMMPS (2024) GROMACS (2023.3) NAMD (3.0)	Palabos (1.4) OpenLB (1.7) LBMflow (1.0.1)	IBAMR (0.14.0) MATLAB (R2023a)

## Abbreviations

2D	Two-dimensional
3D	Three-dimensional
AI	Artificial Intelligence
ATAA	Ascending Thoracic Aortic Aneurysm
BEM	Boundary element method
BGK	Bhathagar–Gross–Krook
CFD	Computational fluid dynamics
CS-FEM	Cell-based Smoothed Finite Element Method
CSM	Computational Structural Mechanics
CT	Computed Tomography
CTCs	Circulating tumour cells
DLD	Deterministic lateral displacement
EM	Electromagnetic
FEM	Finite Element Method
FSI	Fluid–structure interaction
IBM	Immersed boundary method
IPMF	Inertial Particle Microfluidics
LBE	Lattice Boltzmann equation
LBGK	Lattice Bhathagar–Gross–Krook
LBM	Lattice Boltzmann method
LSM	Lattice spring model
MD	Molecular dynamics
MEMS	Micro-electromechanical systems
PDEs	Partial differential equations
PDMS	Polydimethylsiloxane
PMMA	Polymethyl methacrylate
PZT	Piezoelectric
RBCs	Red blood cells
SBB	Simple bounce back
S-FEM	Smoothed Finite Element Methods
SPHs	Smoothed Particle Hydrodynamics
WBCs	White blood cells
